# Supplementary material for: RIP-MD: a tool to study residue interaction networks in protein molecular dynamics
Source: PeerJ. 2018 Dec 7;6:e5998. doi: 10.7717/peerj.5998 (PMC6287582; doi:10.7717/peerj.5998)
Supplement: Supplemental Information 1 [file peerj-06-5998-s001.pdf]

RIP-MD

User Manual

S. Contreras & A.J.M. Martin

scontreras@dlab.cl | alberto.martin@umayor.cl

Contents

1 Introduction **3**
- 1.1 Purpose of RIP-MD . . . 3
- 1.2 What RIP-MD does . . . 3
- 1.3 RIP-MD flavors and availability . . . 4
- 1.4 How it works . . . 4
- 1.5 Citing Us . . . 6

2 Getting and Installing *RIP-MD* **7**
- 2.1 Getting RIP-MD for Linux . . . 7
- 2.2 Basic installation . . . 7
- 2.3 Advanced installation . . . 8
  - 2.3.1 Installing RIP-MD as Standalone software . . . 8
  - 2.3.2 Installing RIP-MD as VMD plugin . . . 9
- 2.4 Getting RIP-MD for Windows and mac OS . . . 11

3 User Interface **11**
- 3.1 Webserver Interface . . . 11
- 3.2 Standalone Interface . . . 11
- 3.3 VMD plugin Interface . . . 14

4 Running a Job **18**
- 4.1 Required Input . . . 18
- 4.2 Running a Job using the Webserver . . . 18
- 4.3 Running a Job using the Standalone software . . . 18
- 4.4 Running a Job using the VMD plugin Interface . . . 22

5 Output Files and Displaying Results **23**
- 5.1 Output Files . . . 23
- 5.2 Displaying Results . . . 25
  - 5.2.1 Using the RIP-MD plugin . . . 25
  - 5.2.2 Using external software . . . 29
- 5.3 Output Analysis example. . . . 29

A Appendix **33**
- A.1 Definition of C_α contacts . . . 33
- A.2 Definition of Hydrogen Bonds . . . 33
- A.3 Definition of Salt Bridges . . . 33

A.4 Definition of Disulfide Bonds 34
A.5 Definition of cation-π and π-π interactions 34
A.6 Definition of arginine-arginine interactions 35
A.7 Definition of Coulomb and van der Waals potential interactions 35

1 Introduction

1.1 Purpose of RIP-MD

To perform their function, proteins adopt a three dimensional structure that is mainly determined by non-covalent interactions between its Amino Acids (AAs). The structure of a protein is not static, residues may undergo conformational rearrangements, and in doing so, create, stabilize or break non-covalent interactions. Molecular Dynamics (MD) is a computational technique widely used to simulate these movements at atomic resolution. However, it is very difficult to gather relevant structural and functional information from MD simulations given the data-intensive nature of the technique. Consequently, several tools are often required to perform these highly complex analyses. Among these are Residue Interaction Networks (RINs), which have been used to facilitate the study of static protein structures. In a RIN, nodes represent AAs and the connections between nodes depict the non-covalent interactions that occur in the three dimensional structure of the protein. For this reason, we created *RIP-MD*, a tool that create RINs for a protein structure.

1.2 What RIP-MD does

RIP-MD generates RINs for static protein structures or from MD trajectory files. The non-covalent interactions defined by RIP-MD include Hydrogen bonds, Salt bridges, Van der Waals contacts, cation-π, π-π, Arginine-Arginine and Coulomb interactions. In addition, RIP-MD also computes interactions based on distances between C_αs and disulphide bridges.
Results of the analysis can be displayed in an user friendly interface. Moreover, the user can take advantage of the VMD visualization capacities, whereby through some effortless steps, it is possible to select and visualize interactions described for a single, several or all residues in a MD trajectory. Network and descriptive table files are also generated, allowing their further study in other specialized platforms. Furthermore RIP-MD generates correlation plots, where relationships between the dynamic behavior of different parts of the protein can be determined and quantified.

1.3 RIP-MD flavors and availability

RIP-MD comes in three different forms. The first one is as a webserver that can be accessed at `http://dlab.cl/ripmd` → "Star a Job" tab. This version was thought for people who wants to analyze a single PDB structure without installing anything in local machines.
If you want to analyze a MD trajectory, or a static structure in a local machine, you can go to `http://dlab.cl/ripmd`, where in the "Download RIP-MD" section you can find the standalone and the VMD plugin version.

1.4 How it works

Basically, RIP-MD performs the workflow shown in fig. 1. Starting from a MD or a PDB file and input parameters (see chapter 4, fig. 1 A) it passes to a preprocessing step (fig. 1 B and 2) where input type, repeated residues and missing atoms are looked for. The next step is to compute interactions between residues (defined in appendix A) and in the last step the output is displayed either with the VMD plugin or on softwares such as Cytoscape[1]

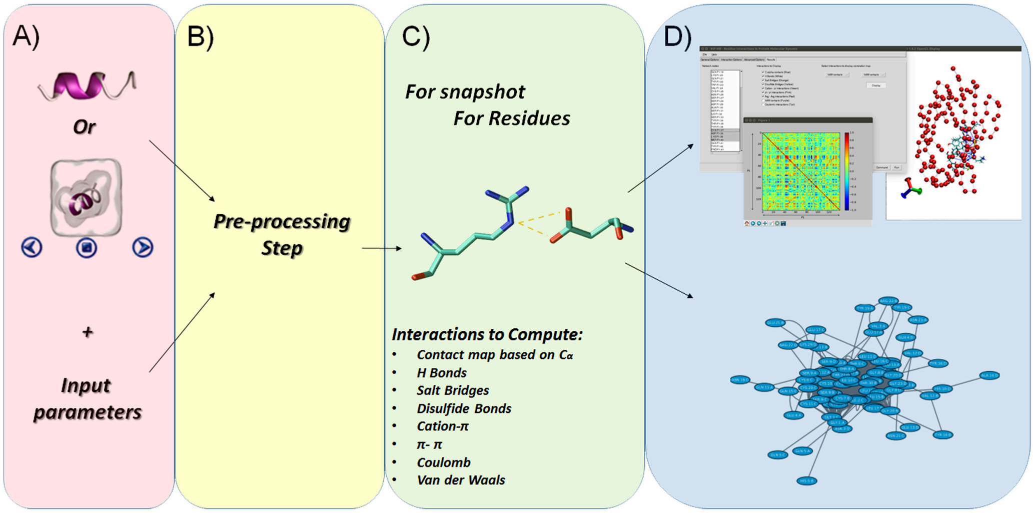

Figure 1: Workflow in *RIP-MD*. A) Input structural information and parameters. B) Pre-processing step. C) Definition of putative interactions as defined by the input parameters. D) Generation of output files.

[1] `www.cytoscape.org`

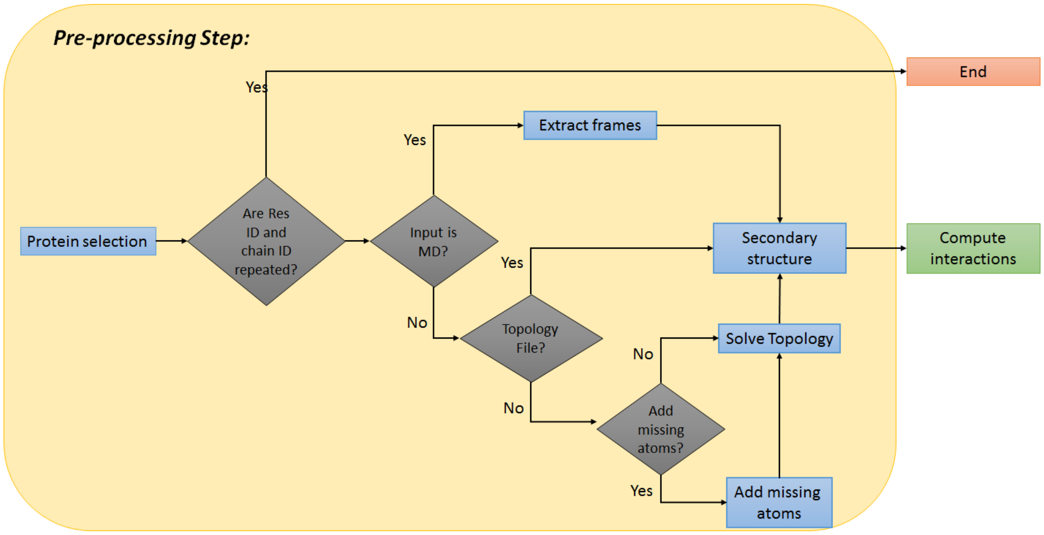

Figure 2: Preprocessing step. Starting with the protein selection all atoms not belonging to an AA are removed, as well as repeated residues. Then, if the structural input is a MD trajectory each frame is extracted, or otherwise a check for the existence of a topology file of the initial PDB is performed. In this last case, if the user wishes it, missing atoms will be added, to end up calculating some AA properties such as secondary structure and SASA.

1.5 Citing Us

At this moment, RIP-MD is under revision, as soon as possible we will add and update the information to cite this work

2 Getting and Installing *RIP-MD*

In order to generate a correct local installation of the software, you need to use it in a machine running a Linux distribution, although we know it may work on Mac Operating System (OS). If your still want to use it in Windows and mac-OS, we recommend to use the virtual machine running Ubuntu available in our web page (`http://dlab.cl/ripmd`). On this virtual machine, RIP-MD is already preinstalled with all its dependencies and you will only need to install VMD. We strongly recommend to use RIP-MD under Debian[2] or Ubuntu[3] Linux distributions since we know it works there. However, if you only want to use the graphical user interface (VMD plugin), you can use it in any system where VMD can be installed, to visualize jobs generated in a Linux machine.
As a second requirement, you need to have *python* version 2.7 or higher to run the program, however it will not run under python3 due to some dependencies not implemented yet in python3.
If you want to use the Plugin user interface, the only additional requirement is to have installed VMD[4] in your system.

2.1 Getting RIP-MD for Linux

You can download RIP-MD from `http://dlab.cl/ripmd`, but keep in mind that we only tested it on Linux machines, although we know it may work on Mac OS. In the "Download RIP-MD" tab you will see a table with links to download the software in all available forms. After you have downloaded RIP-MD, you need to uncompress it.

2.2 Basic installation

To simplify the process of installing RIP-MD, two bash scripts are provided and should be run in this order:

- *requirements.sh*: This script should be executed in sudo mode (using sudo in Ubuntu or changing to the super user using su in Debian). This script looks if python pip is installed and if not, the script will install

[2] `www.debian.org`
[3] `www.ubuntu.com`
[4] `www.ks.uiuc.edu/Research/vmd`

it. Once pip is installed in your system, then the script will read the “libraries” file, and will install all dependencies. If an error occurs in this step, try re-running this script.

- *install.sh*: This script should be executed as normal user. This script will ask you if you want to install the standalone core of RIP-MD, the Plugin version or both and then it will ask the path to where files must be placed.

```
Warning: After the installation process you should close
the terminal (or use another terminal) before use RIP-MD in
order to recognize a new environment variable
```

```
Warning: VMD plugin installation could fail in the last
step (i.e., adding the new plugin to file .vmdrc). If it
happens, an error text will appear at the end of the
installation script, giving instructions on how to fix it
```

2.3 Advanced installation

2.3.1 Installing RIP-MD as Standalone software

In order to install the required dependencies of RIP-MD, the first step is to install *python tk* and *python pip* through the following commands:

```
• apt-get install python-tk
• apt-get install python-pip python-dev build-essential
• pip install –upgrade pip
```

After pip is installed, you need to install some libraries using the following command:

```
pip install -r libraries
```

where "libraries" is each of the files placed in the RIP-MD installation folder. This command will install the following python libraries:

- `biopython v1.70`[a]
- `matplotlib v2.1.0`[b]
- `MDAnalysis v0.16.2`[c]
- `networkx v2.0`[d]
- `numpy v1.13.3`[e]
- `scipy v1.0.0`[f]

[a] www.biopython.org/
[b] www.matplotlib.org
[c] www.mdanalysis.org
[d] networkx.github.io
[e] www.numpy.org
[f] www.scipy.org

When these dependencies are met, you need to copy the "RIP-MD" folder into the folder where indicated by the path where you want to install RIP-MD (e.g.: in your $HOME path). The last step is to create a environment variable in .bashrc file. You can do it using the following command:

```
echo "export RIP_MD=$installationFolder/RIP-MD´´ »
$HOME/.bashrc
```

Where $installationFolder is the path to where RIP-MD folder is located. Then you can run RIP-MD using the following command:

```
python $RIP_MD/main.py
```

2.3.2 Installing RIP-MD as VMD plugin

If you want to install the VMD plugin version of RIP-MD, you need to move the "RIP-MD_vmd´´ folder into VMD plugin path (by default in

/usr/local/lib/vmd/plugin/noarch/tcl/ in Ubuntu/Debian, or in C:\\Program Files (x86)\University of Illinois\VMD\plugins\noarch\tcl in Windows), and after moving it you need to rename it from RIP-MD_vmd to RIP-MD. After these steps you need to modify (as Root) the *.vmdrc* file (usually located in /usr/local/lib/vmd/ in Ubuntu/Debian or in C:\\Program Files (x86)\University of Illinois\VMD\ in Windows) and add the following text to the end of the file:

```
vmd_install_extension ripmd ripmd_tk "Analysis/RIP-MD"
```

Then you can open VMD and in the "Extension" menu, go to Analysis → RIP-MD. If everything was ok, the RIP-MD plugin will appear on your screen.

2.4 Getting RIP-MD for Windows and mac OS

Due some central libraries (for example, MDAnalysis) are not implemented for some OS, in order to run RIP-MD under Windows or mac OS, you need to have installed VirtualBox[5] on your system. VirtualBox is a general-purpose hardware virtualizer, so you can run multiples OS inside your machine. Once you have installed it, you can download an Ubuntu virtual machine with RIP-MD pre-installed from `http://dlab.cl/ripmd` → "Download RIP-MD" tab and then selecting the virtual machine link. After download has finished, you can import the virtual machine to VirtualBox following the steps described in this tutorial `https://docs.oracle.com/cd/E26217_01/E26796/html/qs-import-vm.html`

3 User Interface

This section describes in a simple way the user interface of RIP-MD. Additional information about these interfaces can be found in Section 4.

3.1 Webserver Interface

When you access `http://dlab.cl/ripmd` you will see an interface similar to fig. 3. If you want to start a new job in the web application, you need to navigate to "Start a Job" tab (fig. 3 red dashed box) and then you will see a window similar to figure 4. This page contains a form to select your protein structure (fig. 4 red dashed line), input parameters (fig. 4 blue dashed line), advanced options (fig. 4 yellow dashed line) and a button to start the job (fig. 4 brown dashed line). For more information on how to start a job using the web platform, please refer to section 4.2.

3.2 Standalone Interface

As standalone program, it use the system terminal to execute RIP-MD as can be seen on fig. 5. For usage please refer to section 4.3

[5] `https://www.virtualbox.org/`

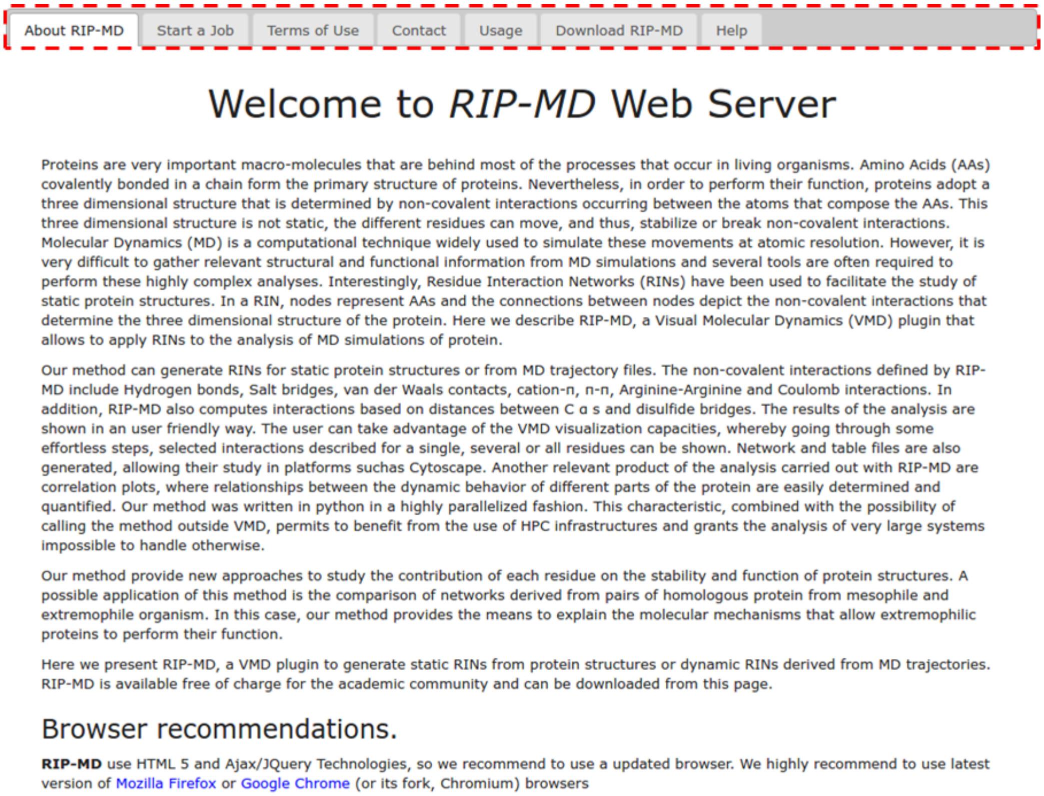

Figure 3: Welcome interface of web version of RIP-MD. The red dashed box highlights the menu to change between different sections of the application.

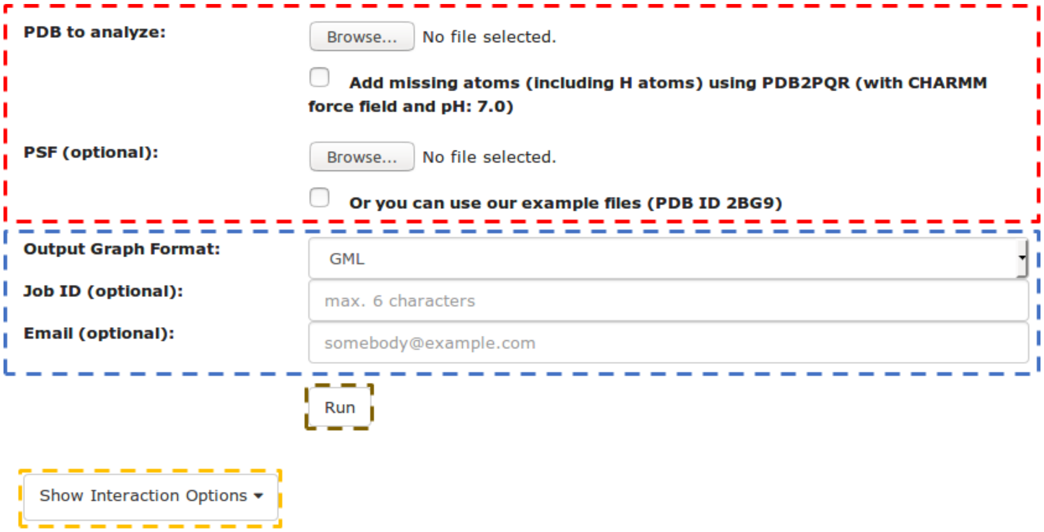

Figure 4: Start job interface of the web application.

```
[redacted]@pc:~$ python $RIP_MD/main.py --help
usage: main.py [-h] [-V] [-D DCD] [-S PSF] [-P PDB] [-sel SELECTION]
               [-O OUTPUT] [-N NPROC] [-ma] [-pH PH] [-ff FORCE_FIELD]
               [-pf PARAMETER_FILE] [-ca] [-cad CA_DIST] [-H] [-hd H_DIST]
               [-ha H_ANGLE] [-s] [-sd S_DISTANCE] [-ss] [-ssd SS_DISTANCE]
               [-ssa SS_ANGLE] [-cp] [-cpd CATION_PI_DISTANCE]
               [-cpa1 CP_ANGLE1] [-cpa2 CP_ANGLE2] [-cpa3 CP_ANGLE3]
               [-cpa4 CP_ANGLE4] [-pp] [-ppd PI_PI_DISTANCE] [-rr]
               [-rrd ARG_ARG_DISTANCE] [-v] [-ve VDW_EXCLUDED] [-vr VDW_RANGE]
               [-c] [-RF] [-Ecs SIMULATED_PERMITTIVITY] [-Erf RF_PERMITTIVITY]
               [-T TEMPERATURE] [-Krf INVERSE_DEBYE_SCREENING]
               [-Rrf DISTANCE_THRESHOLD] [-ce COULOMB_EXCLUDED] [-t TIME]
               [-gf GFORMAT] [-pc] [-p] [-sf SEPARATION_FRAME]
               [-fs FRAME_START] [-fe FRAME_END] [-rf REFERENCE_FRAME]
```

Figure 5: Standalone interface showing all options of RIP-MD.

3.3 VMD plugin Interface

If you installed the plugin, you will see a similar window to that shown in fig 6. In that window you can select and modify some input parameters, such as interactions to be included in the analysis, format for the output and folder where the output will be stored. In this window there is also a tab section to change other parameters. In the “Interaction Options” tab, you will see something like fig. 7, where parameters that define a interaction can be changed. If you click on the “Advanced Options” tab, you will see a window like in fig. 8, where you can select options to add missing atoms, the number of processors to employ, percentage of simulated time to consider interactions to display in the MD consensus graph, and the other parameters. In the “Results” tab, you will see and be able to analyze the results of your analysis. Here, you will also be able to select nodes (AAs) and which interactions will be displayed in VMD or employed to plot Pearson correlations.

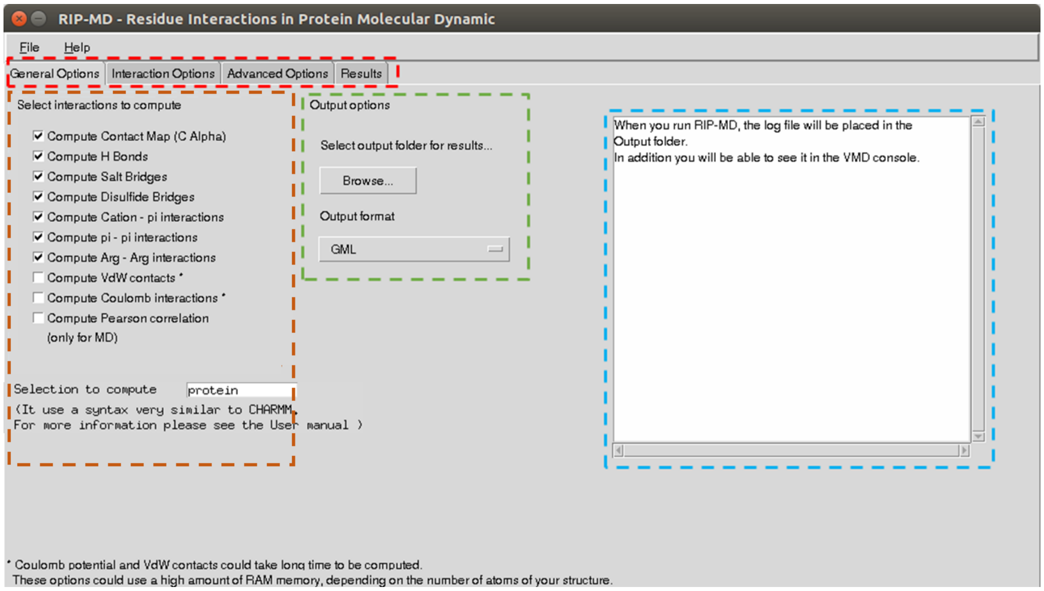

Figure 6: Main interface of RIP-MD. Each colored dashed box represents a distinct element of the software. The red box is the Tab menu of RIP-MD; Brown box is interactions and selection to compute ; Green box: Output options. Light blue box: Output text of the software. Purple box: Buttons to save the command to execute RIP-MD and button to Run directly RIP-MD.

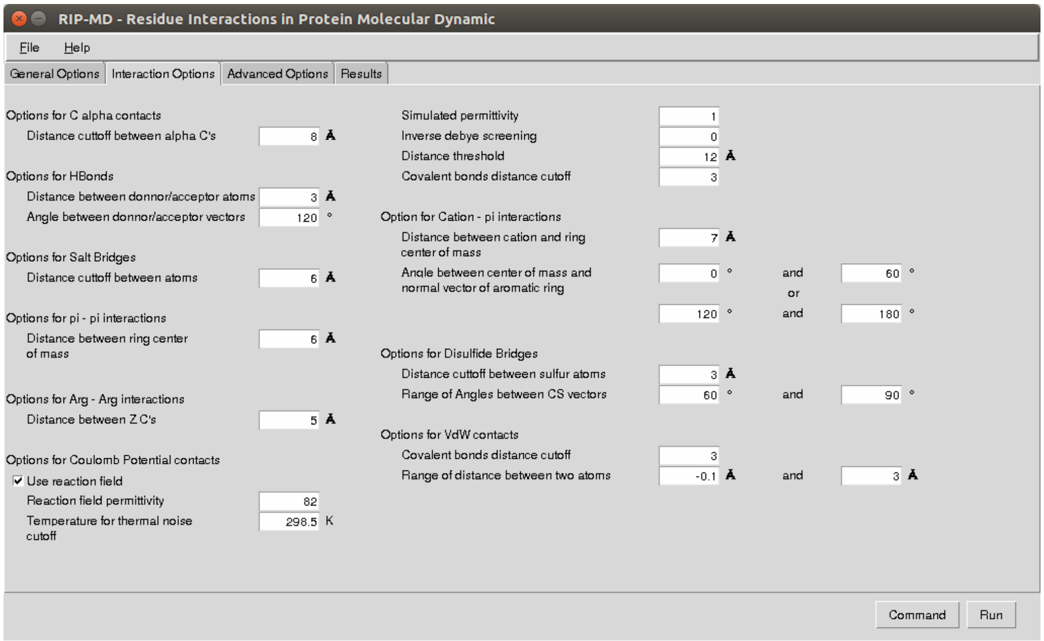

Figure 7: Interaction Options tab. Here you can modify distance, angle, and all parameters employed to define each type of interactions.

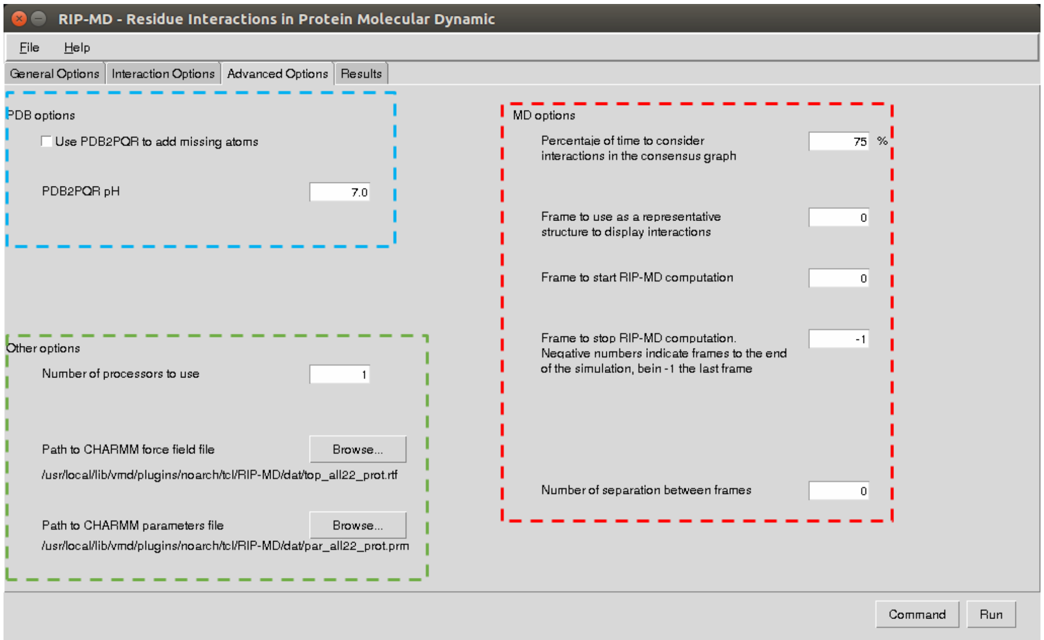

Figure 8: Advanced options of RIP-MD. Light blue dashed box: PDB options, to add missing atoms using *PDB2PQR* software and the pH to compute it. Red dashed box: MD options, to select percentage of simulation time to define interactions that will appear in the consensus graph, frame to use as representative structure and to display results in the VMD interface, and the frame to start and stop the calculation and the separation between frames to analyze. Green dashed box: other options that can be defined by the user (number of processors to use, path to forcefield file and parameter file).

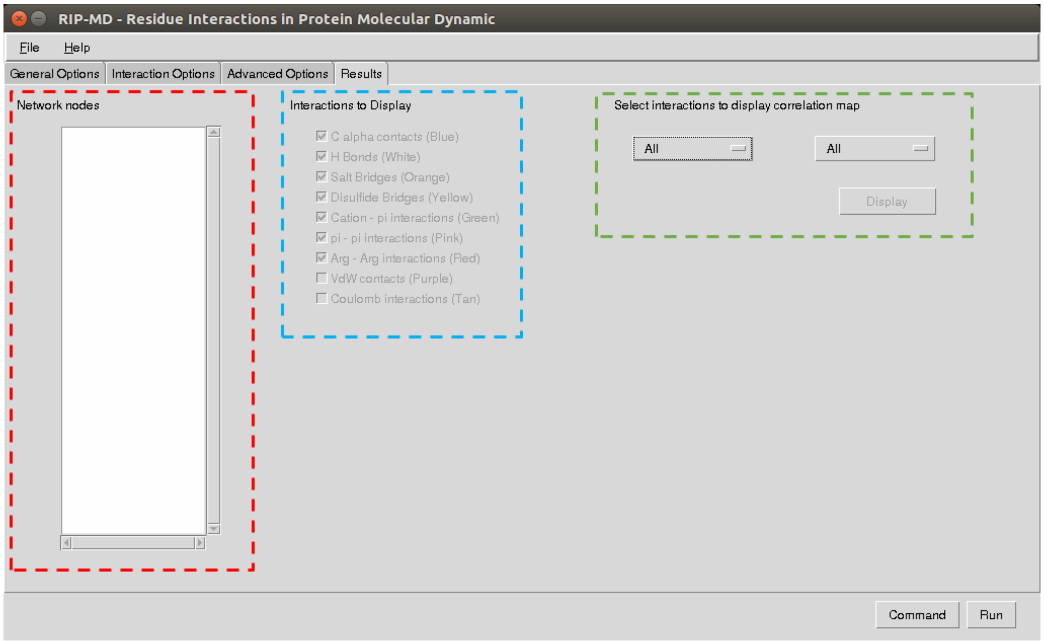

Figure 9: Results tab. Red dashed box: Interface to select nodes to display in the VMD interface. Light blue dashed box: menu to select which interactions you want to see. Green dashed box: selection menu to define the interactions that will be employed to generate Pearson correlation plots.

4 Running a Job

4.1 Required Input

Two class of inputs are supported by RIP-MD. The first class are MD trajectories, using both DCD and PSF files as input. The second class of input are static structures. In this case, RIP-MD requires a PDB file, and optionally a PSF file.
We will perform a little review in the next subsections for the specific parameters for each type of interaction, but for additional information please refer to Appendix A

4.2 Running a Job using the Webserver

In section 3.1 we learn about the interface of the RIP-MD web application in a summarized form. In this section we will explain in a more detailed way how to start a job using this form of RIP-MD.
As can be seen in fig. 4 there are four main sections, where in the red dashed square contains the box to define the input to upload the structural data (a PDB file and an optional PSF). In addition, there are two extra options, the first one is to add missing atoms (including H atoms), and the second option allows to run a job using example files as a way to test what you can do with this software. In the blue dashed square of the fig. 4 you have the two options for the output graph format, a job identifier and your email, both optional. The yellow dashed square of fig. 4, you have a button to display options to change the parameters that define interactions. If you click on it you will see something similar to fig. 10.

When the job finishes, If you provided an email address you are going to receive an email with a link to display your results, however if you do not do it you can save the address in your bookmarks (or wait until job is done) and then download results files.

4.3 Running a Job using the Standalone software

The basic command to start RIP-MD was shown in section 2.3.1. However, all options that can be employed with RIP-MD are displayed with a short description for each option using the follow command in your terminal as

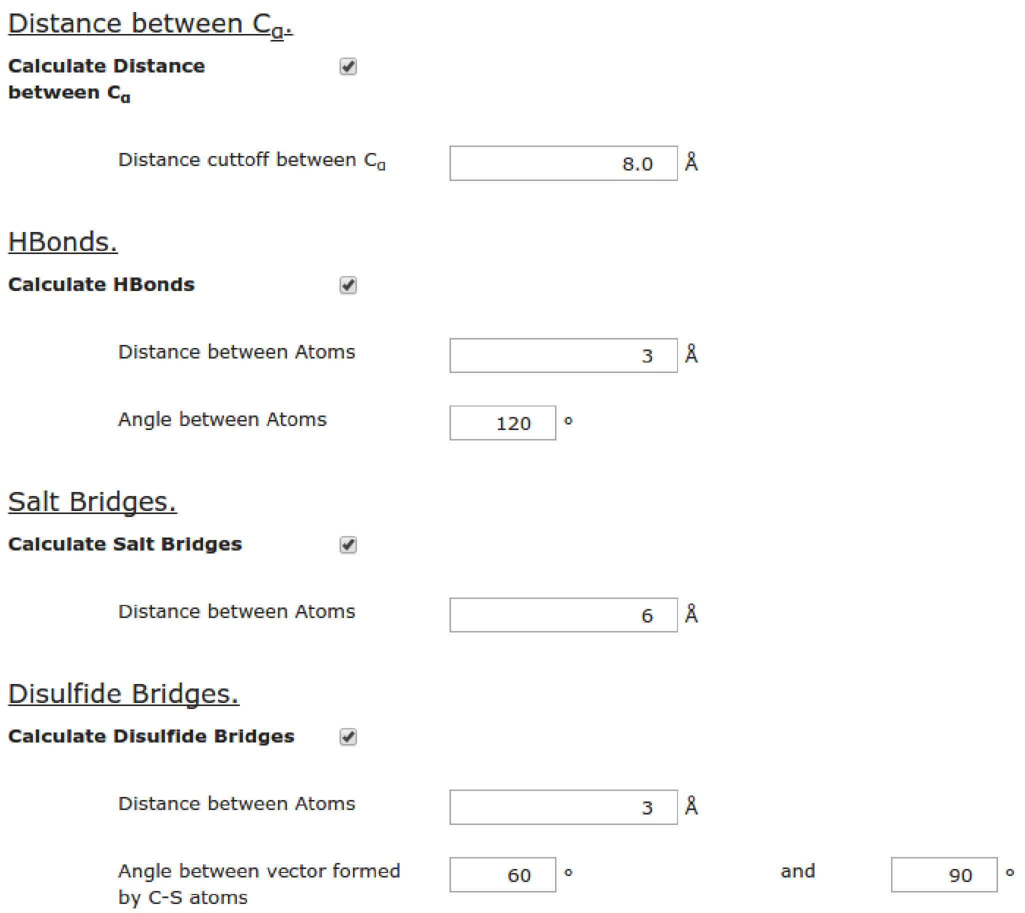

Figure 10: Web interface where the parameters that define interactions can be set.

shown in fig. 5.

```
python $RIP_MD/main.py --help
```

The most basic parameters to run a job are shown in the following text box:

- `-D DCD, --dcd DCD`: Route to the DCD file
- `-S PSF, --psf PSF`: Route to the PSF file
- `-P PDB, --pdb PDB`: Route to the PDB file
- `-sel SELECTION, --selection SELECTION`: Protein selection in CHARMM like format (as defined in MDAnalysis) to perform RINs. Default: "protein"
- `-O OUTPUT, --output OUTPUT`: Path to save results
- `-ca, --calpha`: Calculate C_α contacts
- `-H, --hbond`: Calculate Hbonds
- `-s, --salt`: Calculate Salt bridges
- `-ss, --disulfide`: Calculate disulfide bonds
- `-cp, --cation_pi`: Calculate cation-π interactions
- `-pp, --pi_pi`: Calculate π-π interactions
- `-rr, --arg_arg`: Calculate ARG-ARG interactions
- `-v, --vdw`: Calculate Van deer Waals interaction network
- `-c, --coulomb`: Calculate Coulomb potential between charged groups of each amino acid as defined in the CHARMM force field
- `-pc, --Pearson_corr`: Calculate Pearson Correlation between residue interactions
- `-p, --plot_Pearson`: Generate plots figures for computed correlations

Running RIP-MD in a console may be difficult, nevertheless, using the plugin you can select your desired configuration and save the command to run it. This will be explained in section 4.4

4.4 Running a Job using the VMD plugin Interface

We have already seen in section 3.3 how to use to the plugin and the graphical user interface. So, to compute RINs for your protein, the first thing that you need to do is to load a structure in VMD (PDB, PDB + PSF or DCD and PSF files) then you need to open the plugin and select those interactions together with the protein selection (in CHARMM-like format, for more information please refer to the MDAnalysis web page[6]) that you want to analyze as shown in fig. 6 (brown box). Following that, you need to choose the folder where you want to put the results and the format of the result graphs (fig. 6 green box).

To change any parameter that defines interactions, you can go to the "Interaction Options" tab (fig 7). For more information about the definition of interactions, please refer to appendix A.

Then, in the "Advanced Options tab" you can select the option to add missing atoms (including hydrogens) for a single PDB with *PDB2PQR* and the pH that will be used to do it (fig. 8 light blue box). If you are not working with a static structure, in the MD options section (fig. 8 red box) you can define the percentage of the simulated time to consider an interaction that will appear in the results graph, the frame to use as representative structure to display results in the VMD window, the starting and ending frame to compute RINs (by default first frame or 0 and the last frame or -1, where negative numbers refer to the number of frames until the end of the simulation, being -1 the last frame, -2 the previous frame, and so on) and the number of frames that will be skipped between the starting and ending frame (by default 0).

Other options (fig. 8 green box) are the number of processors to use in the RINs calculation and paths to force field file and the parameter file (by default 1 processor and local CHARMM files in the installation folder).

In the last step, to run the job, you need to click in the "Run" button (fig. 6 purple box). Nevertheless instead of using the Run button, you can use the command button. After doing it a "Save command" screen will appear and you will need to select a folder to save a plain text file with the command to execute the standalone RIP-MD version. With this command you can run the job locally or in another machine.

[6] `https://www.mdanalysis.org/docs/documentation_pages/selections.html`

5 Output Files and Displaying Results

5.1 Output Files

When a new job is going to be executed, RIP-MD asks for an output folder (or a job ID if you are using the web application. This folder will be the root of a directory that will contain the RIP-MD results as can be seen in fig. 11. There you will find two text files (“output.log” and “README”) and a new folder called “RIP-MD_Results”.

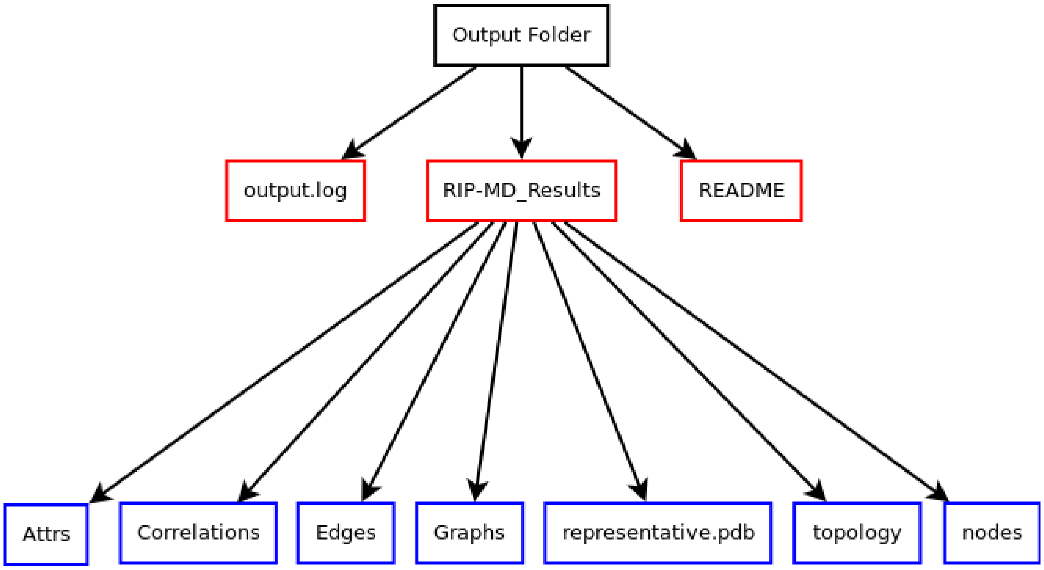

Figure 11: Result folder structure. This folder is divided in three levels (black, red and blue boxes). Output Folder is used to place all created files. In the second level (red boxes), there will be a log file file (output.log), the README file, together with RIP-MD _Results folder. In the third level (blue boxes) there are folders and files created for results.

Next we show a list that summarizes each file/folder that appears in fig. 11

- **output.log**: A simple text file that shows the progress or events that happened during RIP-MD execution.
- **README**: Another text file that explains the components created by the software in the output folder.

- **RIP-MD_Results**: This folder contains the files with the results of RIP-MD
  - **Attrs:** Folder that contains *.attr* file for each snapshot of the MD (or a single if input is a single PDB structure). Each of these files have the attributes of each frame such as secondary structure and SASA.
  - **Correlations:** Folder with Pearson correlations as a text and/or png format if plot_corr option was selected. Only for MD analysis and only if Pearson correlations were computed.
  - **Edges:** Folder that contains *.edge* files for each frame of the MD (or a single if the input is a single PDB structure). Each files present all interactions found.
  - **Graphs:** Folder containing graphs for each interaction computed and a graph containing all interactions (called consensus graph). Supported graph formats are
    - GML
    - EdgeList
    - GraphML
    - Pajek

    Also placed in this folder, there will be a file called "consensus_as_list". This file is used to display interactions in the VMD plugin.
  - **representative.pdb:** A protein structure in *PDB* format that will be used for the VMD plugin to display both: interactions and protein structure.
  - **topology:** Graph file created only if you calculated VDW and/or Coulomb interactions. This file describes the protein topology (data copied from PSF file if it was provided or created *de novo* starting from the protein structure). This file describes data such as VDW radius and bond distances between different atoms.
  - **nodes:** A simple text with node names and IDs for RIP-MD internal usage.

5.2 Displaying Results

5.2.1 Using the RIP-MD plugin

Once a RIP-MD job is finished, you can display the results in the plugin version. If the job run directly in the plugin version, its results will automatically be loaded and they can be analyzed by clicking in the "Results" tab (see fig. 6 red box). If you use the web application or the standalone mode of RIP-MD, you need to go to "File → Open results". Then, you need to open an output folder as shown in fig. 12 (red boxes). After this step, click on the OK button (fig. 12 purple dashed box) to load the results into VMD. Now the result section of RIP-MD will look like in fig. 13 and VMD will load your structure that will look similar to fig. 14.

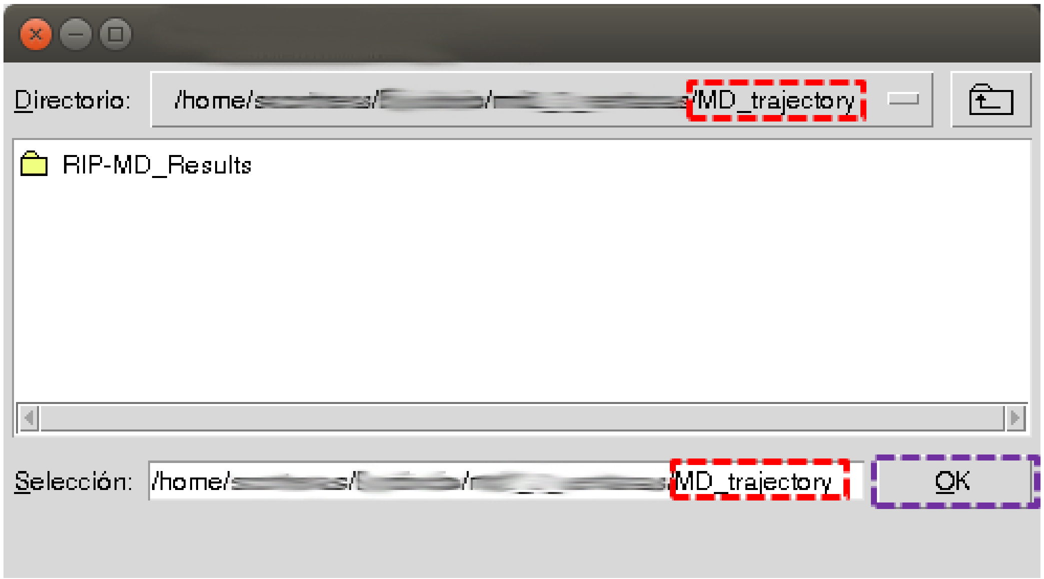

Figure 12: Choose directory window.

To display interaction in the VMD window, you need to select those interactions that you want to analyze, for example C_α contacts you can also select those nodes to study (an example is TRP23 of segment P1 in fig. 13). Now in the VMD you will see interacting amino acids and dashed lines representing interactions like in fig. 15.

In case you want to analyze the correlation between interactions of pairs of residues, you can select this option and then click in the upper "Display"

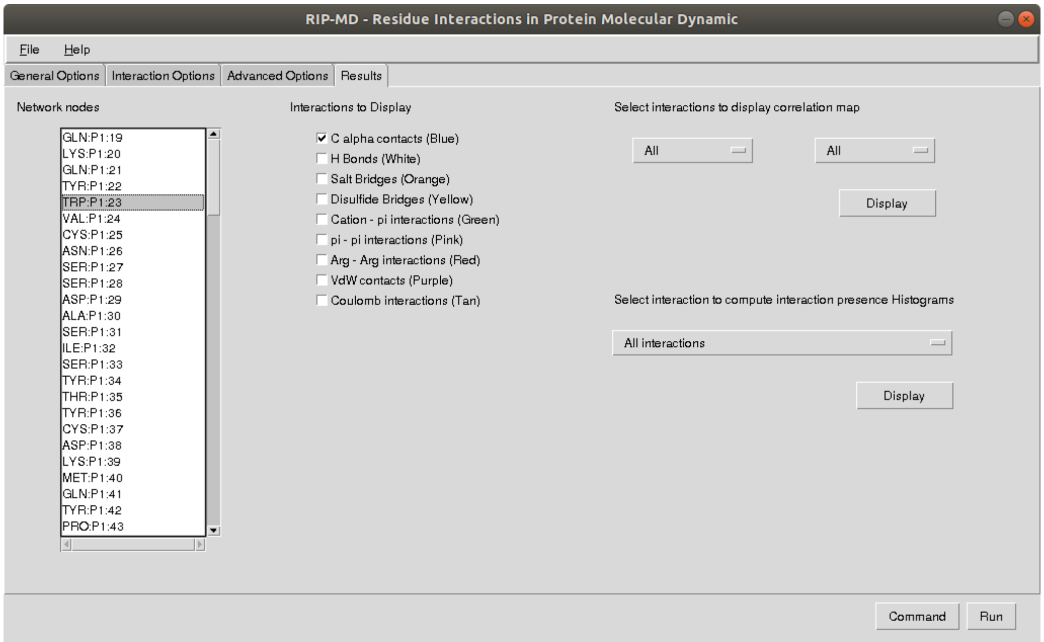

Figure 13: Results loaded in the RIP-MD plugin.

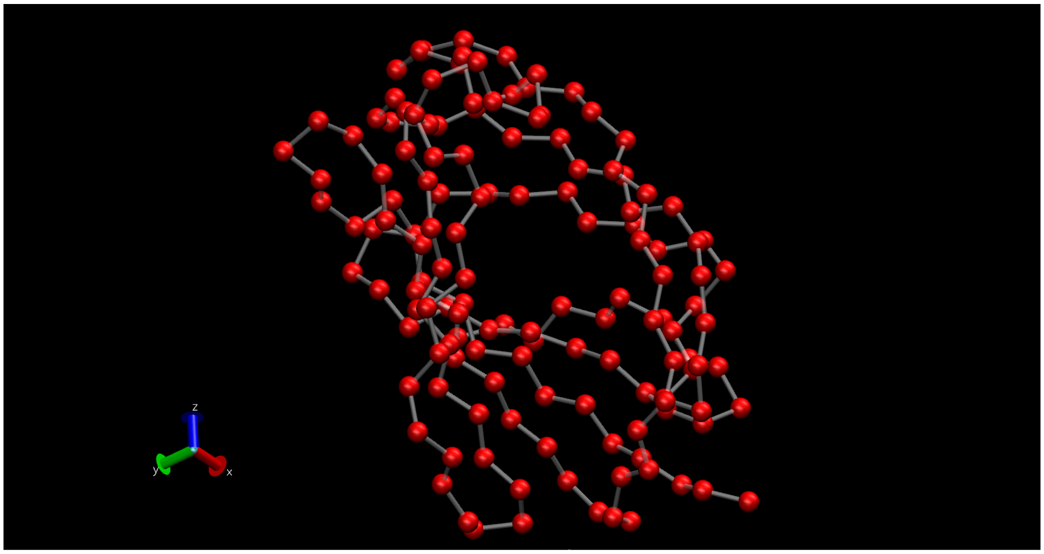

Figure 14: Results loaded in the VMD window. Red balls are amino acids, while gray sticks represent peptidic bonds.

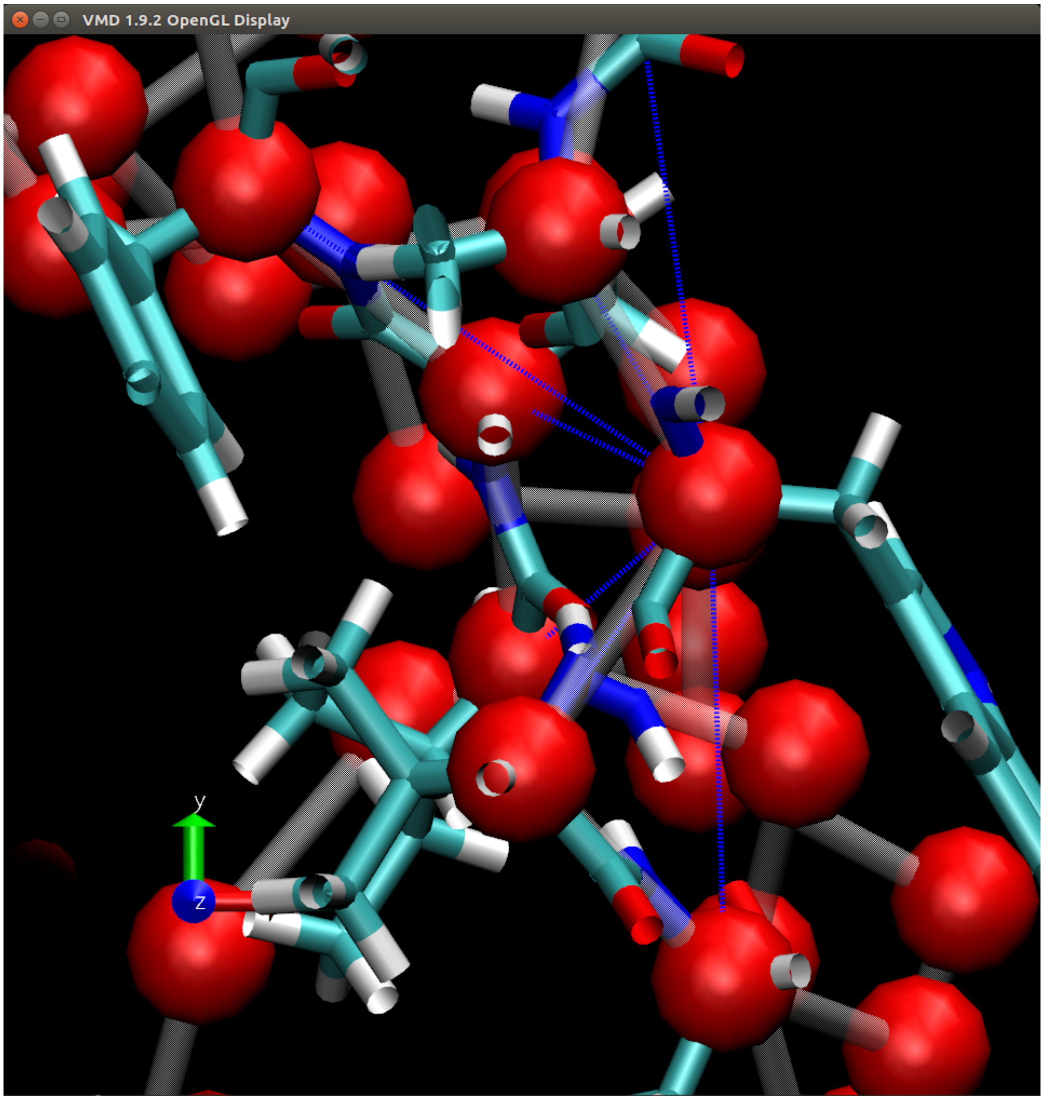

Figure 15: Analyzing interactions in the VMD window. Interacting residues are show as sticks and non-covalent interactions are represented in dashed lines, in this case, C_α contacts.

button (as in fig. 9 green dashed box section). A new window will appear similar to fig. 16 showing the Pearson correlation plot, where you will be able to select determined zones or to save the plot.

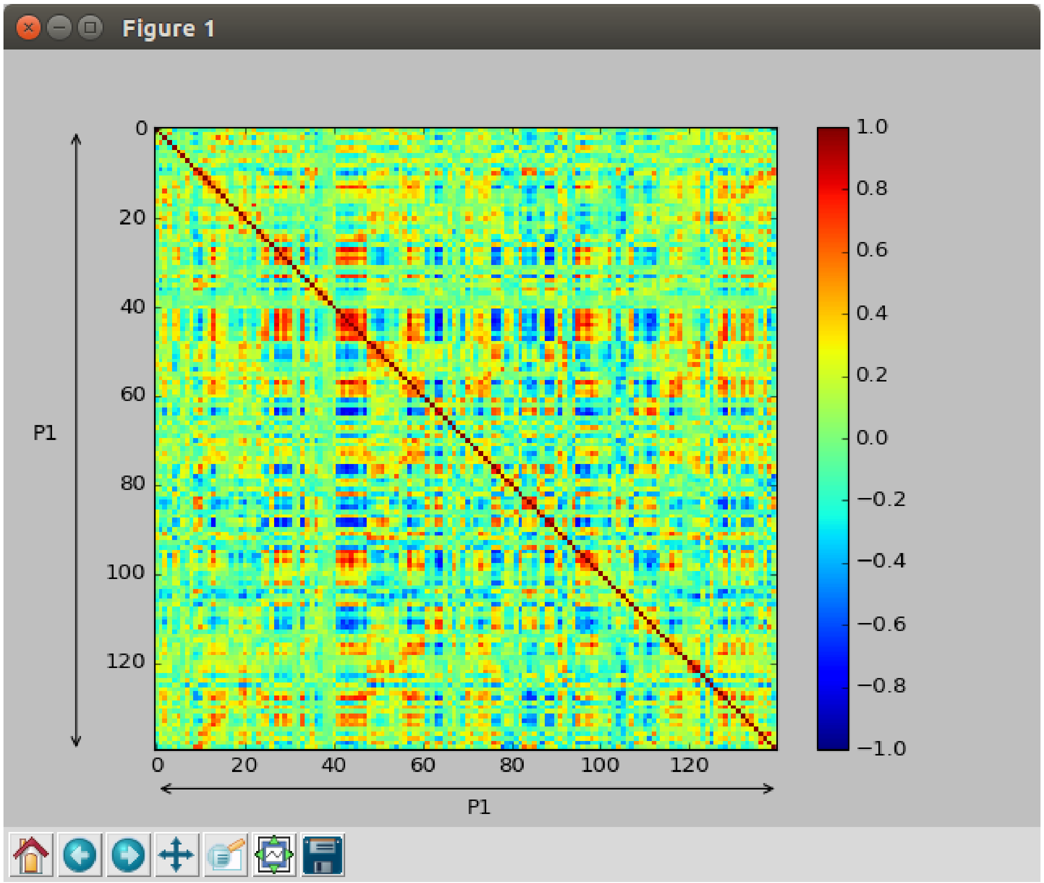

Figure 16: Pearson correlation window generated using RIP-MD. Buttons at the bottom of the window will leave you to select determined zones, modify the figure, restore it or save the image. The color bar at the right side show the color code for different Pearson values.

Another possibility is to visualize the number of interactions present at certain percentage of simulated time. To do it, you can select the type of interaction to analyze in the "Select interactions to generate histograms" section and then click on the "Display" button. After this process is finished, you will see a graphic as the one presented in figure 17.

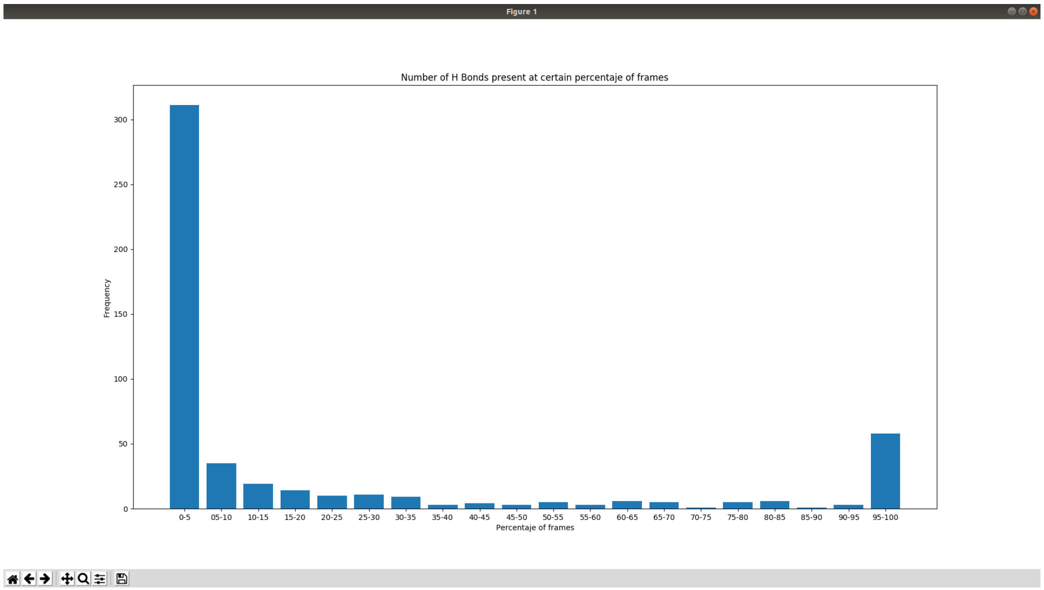

Figure 17: Example of an histogram displaying the number of interactions present at certain each percentage of simulation.

5.2.2 Using external software

If you wish to display RINs in a two dimensions, or if you want to perform some network analysis, you can use Cytoscape to do so. In subsection 5.1 we described the outputs generated by RIP-MD, so you can import into Cytoscape a graph saved in the "Graph" folder. For more information about Cytoscape, please refer to its user manual.

5.3 Output Analysis example.

In order to provide an example, we will use the Nicotinic Acetylcholine Receptor (nAChR), a member of the ligand gated receptor channels family. This protein has five subunits, where the orthosteric site is located in the interface between a subunit α and any other subunit. For this case, we will use the PDB ID 2BG9, a nAChR structure with a resolution of 4Å .

Once the RIP-MD job using this PDB is done (ran with by-default parameters in our example), we will import a graph to analyze it. This is performed by clicking on "File → Import → Network → File" on the plugin interface. A new window will appear similar to Fig. 18, so by navigating it

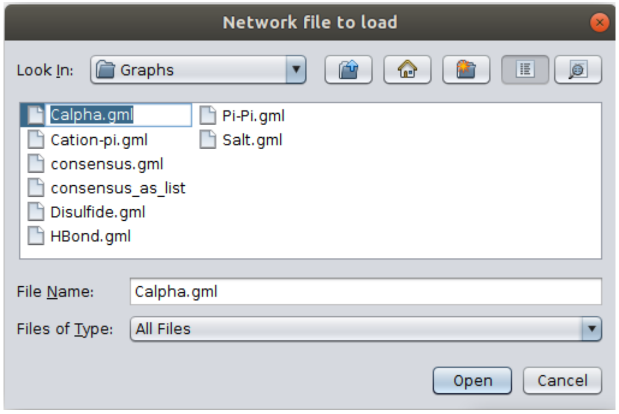

Figure 18: New window to load graph result.

will be able to select the desired graph (for more information about Graphs Files, please refer to section 5.1). In this example we will use C_α contacts graph.

After loading the graph and selecting to display it your preferred layout in Cytoscape, you will see the graph. In the lower part of the screen you will observe a table with information about nodes, so if you select nodes in the graph, the table will change to show you the information related only to your selected nodes.

Many types of centralities can be computed for our network. To do it, you should click on "Tools → NetworkAnalyzer → Network Analysis → Analyze Network". A new window will appear similar to Fig. 19, where you need to select how to treat your network. For our example we are using an undirected network so we choose the second option. In the bottom table new columns will appear with each node centrality values.

The nAChR has two important cysteine residues in each α subunit (Cys 192 and 193) that contribute to the stability of the orthosteric pocket. For this reason we will center this example on these two residues in one chain (chain A). A simple analysis to perform is to observe the node degree (the number of edges that have a node). In our example, Cys 192 has a degree of 4 and Cys 193 a degree of 6. To know which residues are their neighbors, we can do left click in each node → Select → Select First Neighbors. In the following table you can show shared neighbors and for each one.

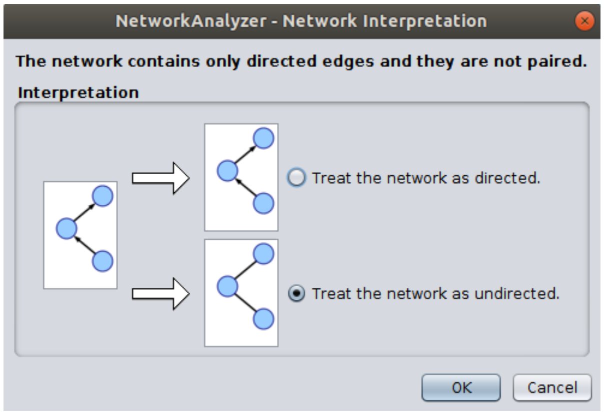

Figure 19: New window where you need to choose your network type.

Type	Nodes
Shared	THR:A:191 - PRO:A:194 - TYR:A:190
Cys 192	CYS:A:193
Cys 193	CYS:A:192 - TYR:A:189 - ASO:A:195

In this way, we will observe that neighbors of these two Cys residues provide a reduced representation of the loop found where the ligand binds to the receptor. To get an overview about how long these residues occupy the acetylcholine pocket, we can use our VMD plugin so it is easy to note that these residues are located in one of the extremes of the loop, but it can not represent the the full interface between both chains for the binding site (Fig 20). This could be explained with additional information about the protein, *i.e.*, the protein is in a closed state, instead of being further away from the second chain as it is in the open state.

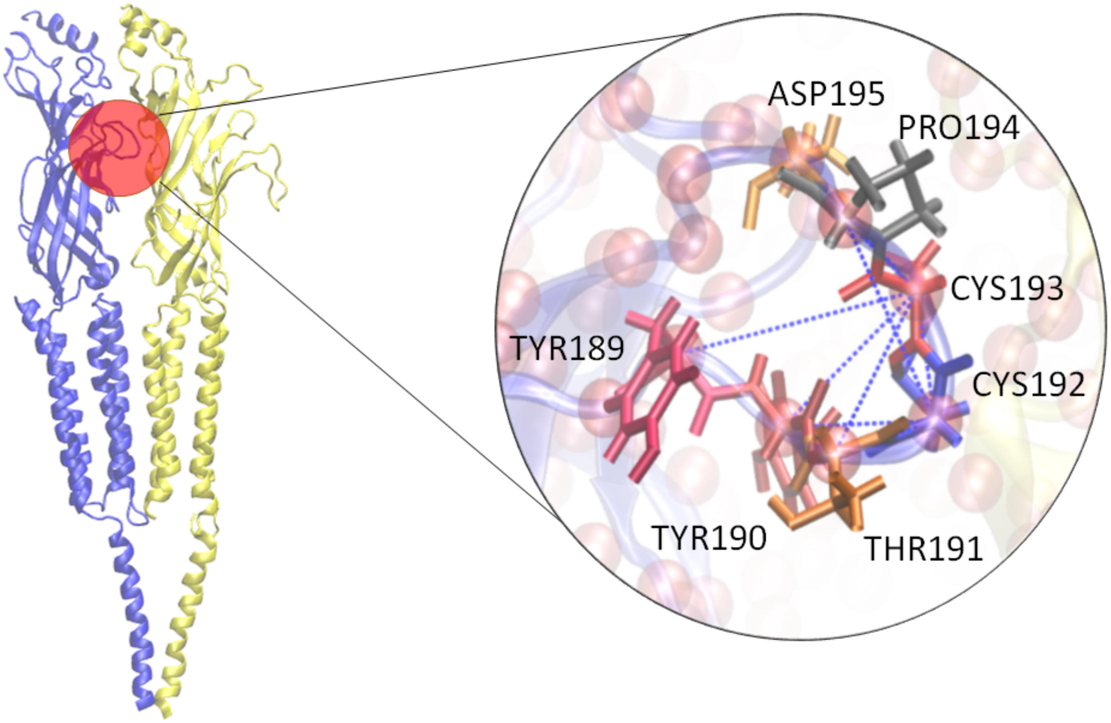

Figure 20: Representation of neighbor residues of Cys192 and Cys193.

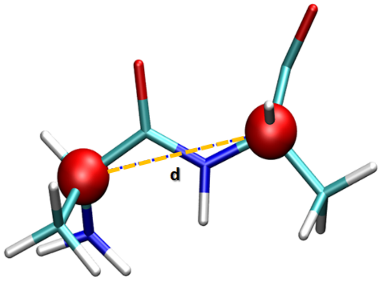

Figure 21: Contact of two C_αs of two different residues. The C_αs are represented as the red spheres and the dashed line represents the contact.

A Appendix

A.1 Definition of C_α contacts

Protein contact maps are described as a binary N x N matrix (where N is the number of AAs or length of the protein). In this matrix, each position i, j is set to 1 if the distance between the C_αs of residues i and j is $\leqslant d$, where d is a distance threshold (Fig. 21) [9, 11, 26]. In RIP-MD the default value of this distance threshold is 8Å[27].

A.2 Definition of Hydrogen Bonds

Hydrogen Bonds (HBs) are identified by a geometric criteria: By default, a pair donor (d) – acceptor (a) is considered to be hydrogen bonded if their distance is less than 3Å and the $\sphericalangle_{cba}$ angle is greater than 120°, with all $\sphericalangle_{cba}$ angles classified as being between 0° and 180° (see Fig. 22.A) [22, 12].

A.3 Definition of Salt Bridges

Salt bridges (SBs) are defined as electrostatic interactions formed between two heavy atoms of opposite charge, where a pair of heavy atoms are within a given distance threshold. In detail, SBs are treated as a contact between NH/NZ in ARG/LYS residues and OE*/OD* in ASP/GLU AAs, and the distance threshold is $\leqslant$ 6Å between those atoms (see Fig. 22.B) [4, 7].

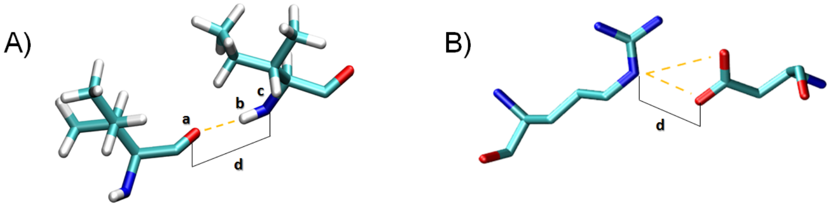

Figure 22: Examples of HBs and SBs. (A) HB defined by the distance d between the two heavy atoms and the angle formed by the electronegative atom (a) and the hydrogen atom (b) covalently bonded to the other electronegative atom (c). (B) SB where d is the distance threshold that defines the interaction.

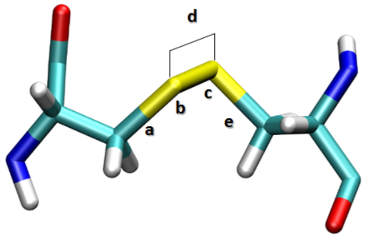

Figure 23: Example of a disulfide bridge between a pair of cysteins. The covalent bonds are defined by dihedral angles for the C-S-S-C atoms ($abce$) between 60° and 90° and a distance between the two sulfur atoms (d) $\leqslant$ 3Å.

A.4 Definition of Disulfide Bonds

Disulfide bonds (DBs) or disulfide bridges are covalent bonds formed between sulfur atoms of the thiol groups of two cystein residues [6, 1, 25]. For static structures *i.e.* a PDB file, *RIP-MD* allows for the definition of DBs employing a geometric criteria: the distance between the sulfur atoms of two cysteins is $\leqslant$ 3Å and the dihedral angle $\measuredangle_{abce}$ is between 60° and 90° (see Fig. 23). Regarding dynamics structures *i.e.* a MD trajectory, we do not provide such capability due to non-reactive nature of common biomolecular force-fields in where all covalent interactions need to be previously established [21, 5, 14].

A.5 Definition of cation-π and π-π interactions

The delocalized nature of π-π bonds in aromatic systems leads to a higher electron density above and below the ring plane. The latter results in particu-

lar electrostatic interaction between π conjugated species and polar moieties, cations, anions or other aromatic rings [2]. Among these, cation-π and π-π are the most abundant and relevant in biological contexts [19]. It is important to mention that in proteins, π-systems are only found in aromatic residues (Phe, Tyr and Trp), while cations involved in this type of interaction belong to Lys and Arg AAs [8, 10].

Cation-π interactions occur between a positively charged ion and the face of an electron-rich π system (see Fig. 24). Histidine residues are a special case in cation-π interactions, as they can act both as a cation or as a π-system depending on their protonation state. In agreement with Liao *et al* [13], *RIP-MD* considers His residues as a cation if they present a protonated nitrogen atom, and as π-system only if they are not protonated. In this way, interactions are defined when an aromatic residue and a charged atom are within a distance threshold $\leqslant$ 7Å. Furthermore, the angle between the vectors formed by the cation and the center of the π system and the normal vector of this π-system must be between 0° and 60° or between 120° and 180° (see Fig. 24.A) [18].

π-π interactions occur between two aromatic rings or π-systems. In *RIP-MD* these are defined considering a distance between the geometric center of each aromatic ring ($\leqslant$ 6Å) (see Fig. 25.A) [18]. *RIP-MD* also computes the orientation of each ring with respect to each other and classifies π-π interactions accordingly, as shown in Fig. 25.B.

A.6 Definition of arginine-arginine interactions

There are several computational studies that have demonstrated that the guanidine group of Arginines can resonate and stabilize an interaction between the side chain of two Args [24, 28]. This interaction between Args can form clusters and is often involved in protein-protein oligomerization, molecular recognition and ligand binding [20]. To detect this type of interaction, *RIP-MD* looks for pairs of Arg residues whose C_ζs are within a distance $\leqslant$ 5Å (see Fig. 26) [17, 20].

A.7 Definition of Coulomb and van der Waals potential interactions

All previously described interactions are a either van der Waals (vdW), electrostatics or combination of these last two. Indeed, from a first-principles

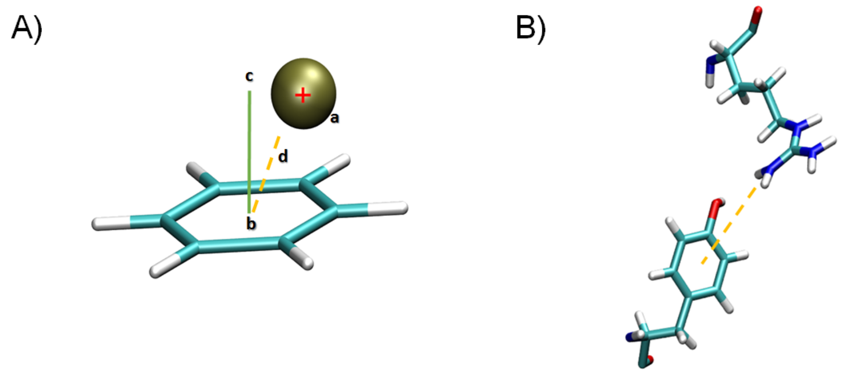

Figure 24: Cation-π interactions. (A) Example of the interaction between a benzene ring and a cation. This interaction is defined by a distance threshold (d) $\leqslant$ 7Å and the $\sphericalangle_{abc}$ between 0° and 60° or between 120° and 180°. (B) Example of the interaction between a Arginine acting as a cation, and a Tyrosine acting as the π-system.

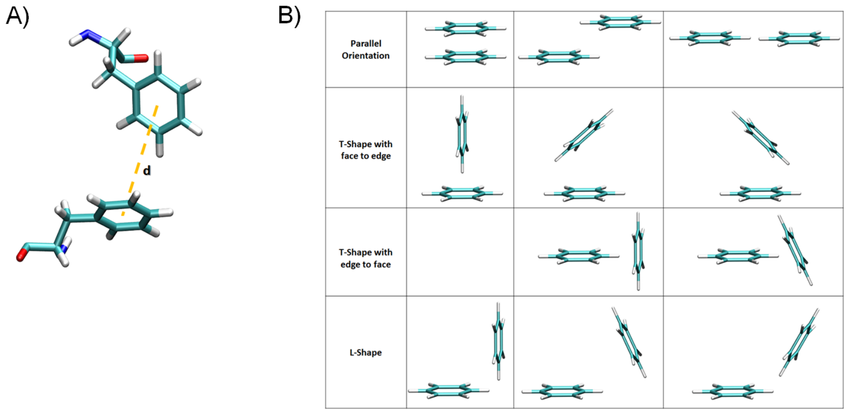

Figure 25: Description of π-π interactions. (A) Example of the interaction between two Phe residues. This interaction exists if the distance between the centroids of the two π-systems (d) is $\leqslant$ 6Å. (B) Definition of the possible orientations of the aromatic rings. These orientations are: Parallel orientation, T-shape with face to edge, T-shape with edge to face and L-shape.

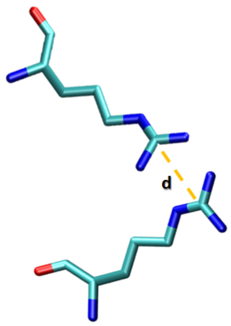

Figure 26: Arginine-Arginine interactions. This interaction is detected when the distance (d) between C_ζ- C_ζ is $\leqslant$ 5Å

.

perspective, all interactions at the molecular level are Coulombic in nature. Thus, inspired by standard MD algorithms[23], *RIP-MD* includes procedures to compute vdW and Coulomb interactions, as it will be explained below.

In *RIP-MD*, vdW contacts are computed using a 12-6 Lennard-Jones potential (V_{LJ}) described by Eq. 1.

$$V_{LJ} = \epsilon \left[\left(\frac{\sigma}{r} \right)^{12} - 2 \left(\frac{\sigma}{r} \right)^{6} \right] \quad (1)$$

where the distance between an atom pair is represented by r, while σ and ϵ respectively are the zero energy distance and the depth of the potential well. These are specific for each atom type, being force-field dependent and are either obtained from when a single structure is provided or the corresponding parameter file if a MD trajectory is submitted to *RIP-MD*.

vdW contacts are defined as shown in Fig. 27. In this figure, atoms 1 and 4 can form a vdW interaction since they are separated by at least three covalent bonds and the distance between the spheres that represent their van der Waals radius (d) is within -0.1Å and 3Å.

To reduce the computational cost caused by the calculation of interactions between each pair of atoms, all coulombic interactions are computed employing a charged-group based cut-off using a 1-4 potential (see Figures 27 and 28) [23, 3, 15, 16]. Thus, interactions between pairs of fully or partially charged atoms pertaining two charged-groups, CG1 and CG2 and within a give cut-off are calculated via:

$$V_{Coulomb} = \frac{1}{4\pi\epsilon_0\epsilon_{cs}} \underbrace{\sum_{i\in \mathrm{CG}_1}\sum_{j\in \mathrm{CG}_2}}_{i,j \text{ not excluded, } j \text{ inside cut-off } i} \frac{q_i q_j}{|\vec{r}_{ij}|} \tag{2}$$

in which ϵ_0 and ϵ_{CS} are the permittivity of vacuum and the simulated media, respectively. Usually, ϵ_{CS} is set to 1 when simulating in explicit solvent conditions. $\vec{r}_{ij}$ is the distance vector between particles i and j. In general MD simulations are run under periodic boundary conditions to avoid surface effects. The latter leads to spurious electrostatics as the $\frac{1}{r}$ does not converge across the box copies. Several strategies exit to alleviate these artifacts, such as lattice sums and the Reaction Field (RF) method. The latter is quite computationally expensive, thus in *RIP-MD* a modified Coulomb potential using the RF formulation is employed. In detail, apart from the so called Coulombic term (see Eq. (2)) two extra terms that mimic the effects of the surrounding solvent are added.

$$V_{DD} = \frac{1}{4\pi\epsilon_0\epsilon_{cs}} \underbrace{\sum_{i\in \mathrm{CG}_1}\sum_{j\in \mathrm{CG}_2}}_{j \text{ inside cut-off } i} \frac{-q_i q_j C_{RF} \vec{r}_{ij}^{\,2}}{2{R_{RF}}^3} \tag{3}$$

and

$$V_{DI} = \frac{1}{4\pi\epsilon_0\epsilon_{cs}} \underbrace{\sum_{i\in \mathrm{CG}_1}\sum_{j\in \mathrm{CG}_2}}_{j \text{ inside cut-off } i} \frac{-q_i q_j (1 - 0.5 C_{RF})}{R_{RF}} \tag{4}$$

with C_{RF} :

$$C_{RF} = \frac{(2\epsilon_{CS} - 2\epsilon_{RF})(1 + \kappa_{RF} R_{RF}) - \epsilon_{RF}(\kappa_{RF} R_{RF})^2}{(\epsilon_{CS} + 2\epsilon_{RF})(1 + \kappa_{RF} R_{RF}) + \epsilon_{RF}(\kappa_{RF} R_{RF})^2} \tag{5}$$

where ϵ_{RF} is the RF permittivity, *i.e.* the permittivity of the solvent, κ_{RF} is the inverse Debye screening length, usually set to 0 for explicit solvent MD and R_{RF} is the RF cut-off. Eqs. (3) and (4) are known as the distance dependent and independent terms, respectively. Eq.(2) is not evaluated for excluded atoms (those connected by 4 or less atoms, *e.g.* a torsion angle), while Eqs. (3) and (4) are evaluated for these atoms, as well.

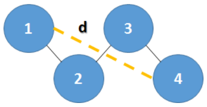

Figure 27: 1-4 Potential for Coulomb and VdW interactions. These interactions are computed if the interacting atoms (atoms 1 and 4) are separated at least by three covalent bonds. Also, the potentials are computed in a distance threshold d.

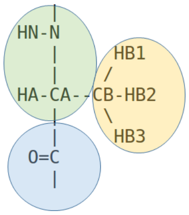

Figure 28: Example of residue charged groups. In this case Alanine is divided in three charged groups (colored circles) as defined by CHARMM topology file.

Given the long-range nature of Eqs. (2), (3) and (4), a raw RIN is rather impractical for visualization purposes. We have adopted two strategies to alleviate this issue: an interaction is considered only if it is above $k_B T$, where k_B is the Boltzmann constant and T is the temperature in kelvin; all interactions are consolidated at the residue level, in other words a node, *i.e.*, an AA, can have multiple edges *i.e.* an interaction, based on it constituent charge-groups (see Fig. 28).

References

[1] D. Alewood, K. Nielsen, P. F. Alewood, D. J. Craik, P. Andrews, M. Nerrie, S. White, T. Domagala, F. Walker, J. Rothacker, A. W. Burgess, and E. C. Nice. The role of disulfide bonds in the structure and function of murine epidermal growth factor (megf). *Growth Factors*, 23(2):97–110, 2005.

[2] E. V. Anslyn and D. A. Dougherty. *Modern Physical Organic Chemistry*, volume 69. 2006.

[3] R. B. Best, X. Zhu, J. Shim, P. E. M. Lopes, J. Mittal, M. Feig, and A. D. MacKerell. Optimization of the additive charmm all-atom protein force field targeting improved sampling of the backbone ϕ, ψ and side-chain $\chi 1$ and $\chi 2$ dihedral angles. *Journal of Chemical Theory and Computation*, 8(9):3257–3273, 2012. PMID: 23341755.

[4] H. R. Bosshard, D. N. Marti, and I. Jelesarov. Protein stabilization by salt bridges: concepts, experimental approaches and clarification of some misunderstandings. *Journal of Molecular Recognition*, 17(1):1–16, 2004.

[5] W. D. Cornell, P. Cieplak, C. I. Bayly, I. R. Gould, K. M. Merz, D. M. Ferguson, D. C. Spellmeyer, T. Fox, J. W. Caldwell, and P. A. Kollman. A second generation force field for the simulation of proteins, nucleic acids, and organic molecules. *Journal of the American Chemical Society*, 117(19):5179–5197, 1995.

[6] A. A. Dombkowski, K. Z. Sultana, and D. B. Craig. Protein disulfide engineering. *{FEBS} Letters*, 588(2):206 – 212, 2014. Protein Engineering.

[7] J. E. Donald, D. W. Kulp, and W. F. DeGrado. Salt bridges: Geometrically specific, designable interactions. *Proteins: Structure, Function, and Bioinformatics*, 79(3):898–915, 2011.

[8] D. A. Dougherty. Cation$-\pi$ interactions involving aromatic amino acids. *The Journal of Nutrition*, 137(6):1504S–1508S, 2007.

[9] J. Eargle and Z. Luthey-Schulten. NetworkView: 3D display and analysis of protein·RNA interaction networks. *Bioinformatics (Oxford, England)*, 28(22):3000–1, nov 2012.

[10] J. P. Gallivan and D. A. Dougherty. Cation-π interactions in structural biology. *Proceedings of the National Academy of Sciences*, 96(17):9459–9464, 1999.

[11] J. Glasgow, T. Kuo, and J. Davies. Protein structure from contact maps: A case-based reasoning Approach. *Information Systems Frontiers*, 8(1):29–36, 2006.

[12] P. A. Hunt, C. R. Ashworth, and R. P. Matthews. Hydrogen bonding in ionic liquids. *Chem. Soc. Rev.*, 44:1257–1288, 2015.

[13] S.-M. Liao, Q.-S. Du, J.-Z. Meng, Z.-W. Pang, and R.-B. Huang. The multiple roles of histidine in protein interactions. *Chemistry Central Journal*, 7(1):1–12, 2013.

[14] A. MacKerell, D. Bashford, M. Bellot, R. Dunbrack, J. Evanseck, M. Field, S. Fischer, J. Gao, H. Guo, S. Ha, D. Joseph-McCarthy, L. Kuchnir, K. Kuczera, F. Lau, C. Mattos, S. Michnick, T. Ngo, D. Nguyen, B. Prodhom, W. Reiher, B. Roux, M. Schlenkrich, J. Smith, R. Stote, J. Straub, M. Watanabe, J. Wiorkiewicz-Kuczera, D. Yin, and M. Karplus. All-Atom Empirical Potential for Molecular Modeling and Dynamics Studies of Proteins. *Journal of Physical Chemistry B*, 102(18):3586–3616, 1998.

[15] A. D. MacKerell, D. Bashford, M. Bellott, R. L. Dunbrack, J. D. Evanseck, M. J. Field, S. Fischer, J. Gao, H. Guo, S. Ha, D. Joseph-McCarthy, L. Kuchnir, K. Kuczera, F. T. K. Lau, C. Mattos, S. Michnick, T. Ngo, D. T. Nguyen, B. Prodhom, W. E. Reiher, B. Roux, M. Schlenkrich, J. C. Smith, R. Stote, J. Straub, M. Watanabe, J. Wiórkiewicz-Kuczera, D. Yin, and M. Karplus. All-atom empirical potential for molecular modeling and dynamics studies of proteins. *The Journal of Physical Chemistry B*, 102(18):3586–3616, 1998. PMID: 24889800.

[16] A. D. MacKerell, M. Feig, and C. L. Brooks. Improved treatment of the protein backbone in empirical force fields. *Journal of the American Chemical Society*, 126(3):698–699, 2004. PMID: 14733527.

[17] A. Magalhaes, B. Maigret, J. Hoflack, J. N. F. Gomes, and H. A. Scheraga. Contribution of unusual arginine-arginine short-range interactions to stabilization and recognition in proteins. *Journal of Protein Chemistry*, 13(2):195–215, 1994.

[18] A. J. M. Martin, M. Vidotto, F. Boscariol, T. Di Domenico, I. Walsh, and S. C. E. Tosatto. RING: networking interacting residues, evolutionary information and energetics in protein structures. *Bioinformatics (Oxford, England)*, 27(14):2003–5, July 2011.

[19] E. A. Meyer, R. K. Castellano, and F. Diederich. Interactions with aromatic rings in chemical and biological recognition, 2003.

[20] M. A. C. Neves, M. Yeager, and R. Abagyan. Unusual arginine formations in protein function and assembly: Rings, strings, and stacks. *The Journal of Physical Chemistry B*, 116(23):7006–7013, 2012. PMID: 22497303.

[21] C. Oostenbrink, A. Villa, A. E. Mark, and W. F. van Gunsteren. A biomolecular force field based on the free enthalpy of hydration and solvation: the GROMOS force-field parameter sets 53A5 and 53A6. *Journal of computational chemistry*, 25(13):1656–76, Oct. 2004.

[22] T. Steiner. The hydrogen bond in the solid state. *Angewandte Chemie International Edition*, 41(1):48–76, 2002.

[23] W. van Gunsteren, P. Hünenberger, A. Mark, P. Smith, and I. Tironi. Computer simulation of protein motion. *Computer Physics Communications*, 91(1-3):305–319, Sept. 1995.

[24] J. Vondrášek, P. E. Mason, J. Heyda, K. D. Collins, and P. Jungwirth. The molecular origin of like-charge arginine-arginine pairing in water. *The Journal of Physical Chemistry B*, 113(27):9041–9045, 2009. PMID: 19354258.

[25] J. Wang, H. Ma, X. Ge, J. Zhang, K. Teng, Z. Sun, and J. Zhong. Bovicin hj50-like lantibiotics, a novel subgroup of lantibiotics featured by an indispensable disulfide bridge. *PLoS ONE*, 9(5):1–11, 05 2014.

[26] Z. Wang and J. Xu. Predicting protein contact map using evolutionary and physical constraints by integer programming. *Bioinformatics*, 29(13):266–273, 2013.

[27] Z. Wang and J. Xu. Predicting protein contact map using evolutionary and physical constraints by integer programming. *Bioinformatics*, 29(13):i266, 2013.

[28] M. L. Williams and J. E. Gready. Guanidinium-type resonance stabilization and its biological implications. i. the guanidine and extended-guanidine series. *Journal of Computational Chemistry*, 10(1):35–54, 1989.
