# Supplementary material for: RIP-MD: a tool to study residue interaction networks in protein molecular dynamics
Source: PeerJ. 2018 Dec 7;6:e5998. doi: 10.7717/peerj.5998 (PMC6287582; doi:10.7717/peerj.5998)
Supplement: Supplemental Information 2 [file peerj-06-5998-s002.pdf]

RIP-MD:
A tool to study Residue Interaction Networks in Protein Molecular Dynamics: Text S1

Sebastian Contreras-Riquelme, Jose-Antonio Garate, Tomas Perez-Acle & Alberto J.M. Martin*
* *alberto.martin@mayor.cl*

1 Interactions defined in RIP-MD

1.1 Cα based contacts:

Protein contact maps are described as a binary N x N matrix (where N is the number of Amino Acids (AAs) or length of the protein). In this matrix, each position i, j is set to 1 if the distance between the C_αs of residues i and j is $\leqslant d$, where d is a distance threshold (Fig. 1) [11, 13, 32]. In RIP-MD the default value of this distance threshold is 8Å[33].

1.2 Hydrogen Bonds:

Hydrogen Bonds (HBs) are identified by a geometric criteria: By default, a pair donor (d) – acceptor (a) is considered to be hydrogen bonded if their distance is less than 3Å and the $\sphericalangle_{cba}$ angle is greater than 120°, with all $\sphericalangle_{cba}$ angles classified as being between 0° and 180° (see Fig. 2.A) [28, 15].

1.3 Salt Bridges:

Salt bridges (SBs) are defined as electrostatic interactions formed between two heavy atoms of opposite charge, where a pair of heavy atoms are within a given

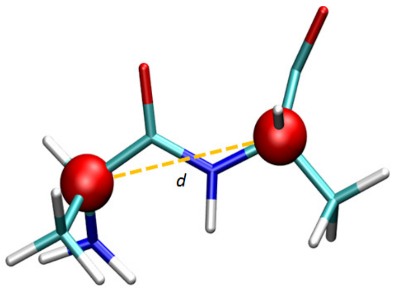

Figure 1: Contact of two C_αs of two different residues. The C_αs are represented as the red spheres and the dashed line represents the contact.

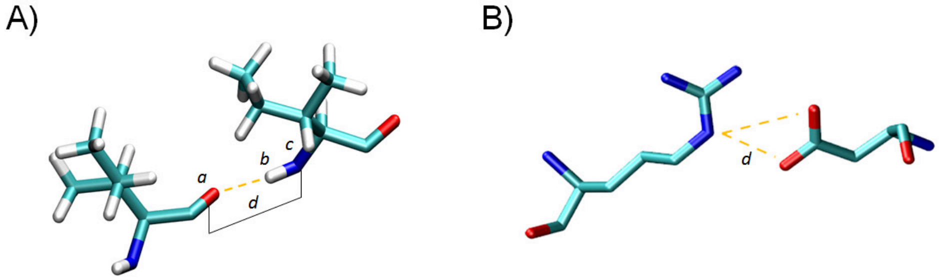

Figure 2: Examples of HBs and SBs. (A) HB defined by the distance d between the two heavy atoms and the angle formed by the electronegative atom (a) and the hydrogen atom (b) covalently bonded to the other electronegative atom (c). (B) SB where d is the distance threshold that defines the interaction.

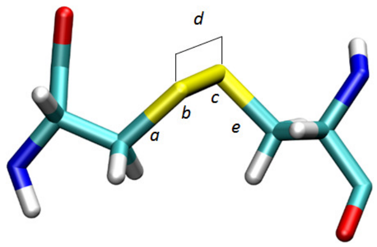

Figure 3: Example of a disulfide bridge between a pair of cysteins. The covalent bonds are defined by dihedral angles for the C-S-S-C atoms ($abce$) between 60° and 90° and a distance between the two sulfur atoms (d) $\leqslant$ 3Å.

distance threshold. In detail, SBs are treated as a contact between NH/NZ in ARG/LYS residues and OE*/OD* in ASP/GLU AAs, and the distance threshold is $\leqslant$ 6Å between those atoms (see Fig. 2.B) [5, 9].

1.4 Disulfide bonds:

Disulfide bonds (DBs) or disulfide bridges are covalent bonds formed between sulfur atoms of the thiol groups of two cystein residues [8, 2, 31]. For static structures *i.e.* a PDB file, *RIP-MD* allows for the definition of DBs employing a geometric criteria: the distance between the sulfur atoms of two cysteins is $\leqslant$ 3Å and the dihedral angle $\sphericalangle_{abce}$ is between 60° and 90° (see Fig. 3). Regarding dynamics structures *i.e.* a Molecular Dynamics (MD) trajectory, we do not provide such capability due to non-reactive nature of common biomolecular force-fields in where all covalent interactions need to be previously established [26, 7, 17].

1.5 Cation-π and π-π interactions:

The delocalized nature of π-π bonds in aromatic systems leads to a higher electron density above and below the ring plane. The latter results in particular electrostatic interaction between π conjugated species and polar moieties,

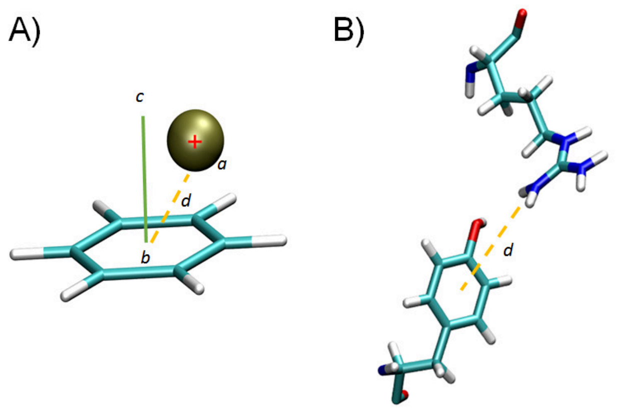

Figure 4: Cation-π interactions. (A) Example of the interaction between a benzene ring and a cation. This interaction is defined by a distance threshold (d) $\leqslant$ 7Å and the $\sphericalangle_{abc}$ between 0° and 60° or between 120° and 180°. (B) Example of the interaction between a Arginine acting as a cation, and a Tyrosine acting as the π-system.

cations, anions or other aromatic rings [3]. Among these, cation-π and π-π are the most abundant and relevant in biological contexts [23]. It is important to mention that in proteins, π-systems are only found in aromatic residues (Phe, Tyr and Trp), while cations involved in this type of interaction belong to Lys and Arg AAs [10, 12].

Cation-π interactions occur between a positively charged ion and the face of an electron-rich π system (see Fig. 4). Histidine residues are a special case in cation-π interactions, as they can act both as a cation or as a π-system depending on their protonation state. In agreement with Liao *et al* [16], *RIP-MD* considers His residues as a cation if they present a protonated nitrogen atom, and as π-system only if they are not protonated. In this way, interactions are defined when an aromatic residue and a charged atom are within a distance threshold $\leqslant$ 7Å. Furthermore, the angle between the vectors formed by the cation and the center of the π system and the normal vector of this π-system must be between 0° and 60° or between 120° and 180° (see Fig. 4.A) [21].

π-π interactions occur between two aromatic rings or π-systems. In *RIP-MD* these are defined considering a distance between the geometric center of each aromatic ring ($\leqslant$ 6Å) (see Fig. 5.A) [21]. *RIP-MD* also computes the orientation of each ring with respect to each other and classifies π-π interactions accordingly, as shown in Fig. 5.B.

1.6 Arg-Arg interactions:

There are several computational studies that have demonstrated that the guanidine group of Arginines can resonate and stabilize an interaction between the side chain of two Args [30, 34]. This interaction between Args can form clusters and is often involved in protein-protein oligomerization, molecular recognition and ligand binding [25]. To detect this type of interaction, *RIP-MD* looks for pairs of Arg residues whose C_ζs are within a distance $\leqslant$ 5Å (see Fig. 6) [20, 25].

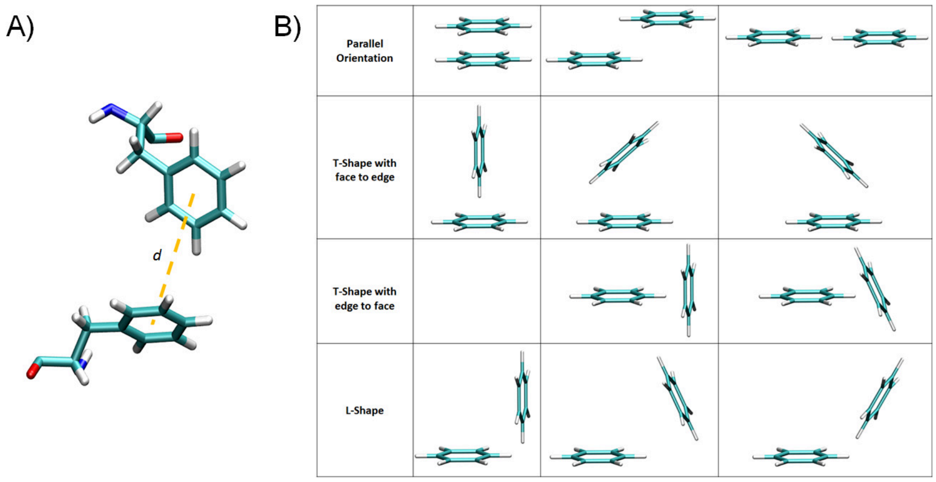

Figure 5: Description of π-π interactions. (A) Example of the interaction between two Phe residues. This interaction exists if the distance between the centroids of the two π-systems (d) is $\leqslant$ 6Å. (B) Definition of the possible orientations of the aromatic rings. These orientations are: Parallel orientation, T-shape with face to edge, T-shape with edge to face and L-shape.

1.7 Coulomb interactions and van der Waals (vdW) contacts:

All previously described interactions are a either vdW, electrostatics or combination of these last two. Indeed, from a first-principles perspective, all interactions at the molecular level are Coulombic in nature. Thus, inspired by standard MD algorithms[29], *RIP-MD* includes procedures to compute vdW and Coulomb interactions, as it will be explained below.

In *RIP-MD*, vdW contacts are computed using a 12-6 Lennard-Jones potential (V_{LJ}) described by Eq. 1.

$$V_{LJ} = \epsilon \left[\left(\frac{\sigma}{r} \right)^{12} - 2 \left(\frac{\sigma}{r} \right)^{6} \right] \tag{1}$$

where the distance between an atom pair is represented by r, while σ and ϵ respectively are the zero energy distance and the depth of the potential well. These are specific for each atom type, being force-field dependent and are either obtained from when a single structure is provided or the corresponding parameter file if a MD trajectory is submitted to *RIP-MD*.

vdW contacts are defined as shown in Fig. 7. In this figure, atoms 1 and 4 can form a vdW interaction since they are separated by at least three covalent bonds and the distance between the spheres that represent their van der Waals radius (d) is within -0.1Å and 3Å.

To reduce the computational cost caused by the calculation of interactions between each pair of atoms, all coulombic interactions are computed employing a charged-group based cut-off using a 1-4 potential (see Figures 7 and 8) [29,

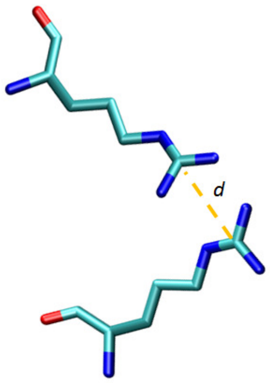

Figure 6: Arginine-Arginine interactions. This interaction is detected when the distance (d) between C_ζ- C_ζ is $\leqslant 5$Å

.

4, 18, 19]. Thus, interactions between pairs of fully or partially charged atoms pertaining two charged-groups, CG1 and CG2 and within a give cut-off are calculated via:

$$V_{Coulomb} = \frac{1}{4\pi\epsilon_0\epsilon_{cs}} \underbrace{\sum_{i\in\mathrm{CG}_1}\sum_{j\in\mathrm{CG}_2}}_{i,j \text{ not excluded, } j \text{ inside cut-off } i} \frac{q_i q_j}{|\vec{r}_{ij}|} \tag{2}$$

in which ϵ_0 and ϵ_{CS} are the permittivity of vacuum and the simulated media, respectively. Usually, ϵ_{CS} is set to 1 when simulating in explicit solvent conditions. $\vec{r}_{ij}$ is the distance vector between particles i and j. In general MD simulations are run under periodic boundary conditions to avoid surface effects. The latter leads to spurious electrostatics as the $\frac{1}{r}$ does not converge across the box copies. Several strategies exit to alleviate these artifacts, such as lattice sums and the Reaction Field (RF) method. The latter is quite computationally expensive, thus in *RIP-MD* a modified Coulomb potential using the RF formulation is employed. In detail, apart from the so called Coulombic term (see Eq. (2)) two extra terms that mimic the effects of the surrounding solvent are added.

$$V_{DD} = \frac{1}{4\pi\epsilon_0\epsilon_{cs}} \underbrace{\sum_{i\in\mathrm{CG}_1}\sum_{j\in\mathrm{CG}_2}}_{j \text{ inside cut-off } i} \frac{-q_i q_j C_{RF} \vec{r}_{ij}^{\,2}}{2{R_{RF}}^3} \tag{3}$$

and

$$V_{DI} = \frac{1}{4\pi\epsilon_0\epsilon_{cs}} \underbrace{\sum_{i\in\mathrm{CG}_1}\sum_{j\in\mathrm{CG}_2}}_{j \text{ inside cut-off } i} \frac{-q_i q_j (1 - 0.5 C_{RF})}{R_{RF}} \tag{4}$$

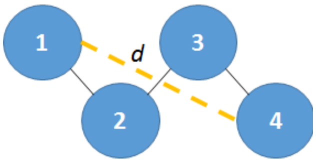

Figure 7: 1-4 Potential for Coulomb and VdW interactions. These interactions are computed if the interacting atoms (atoms 1 and 4) are separated at least by three covalent bonds. Also, the potentials are computed in a distance threshold *d*.

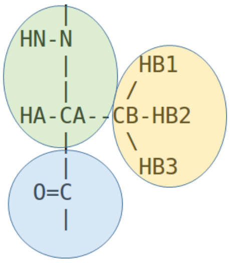

Figure 8: Example of residue charged groups. In this case Alanine is divided in three charged groups (colored circles) as defined by CHARMM topology file.

with C_{RF} :

$$C_{RF} = \frac{(2\epsilon_{CS} - 2\epsilon_{RF})(1 + \kappa_{RF} R_{RF}) - \epsilon_{RF}(\kappa_{RF} R_{RF})^2}{(\epsilon_{CS} + 2\epsilon_{RF})(1 + \kappa_{RF} R_{RF}) + \epsilon_{RF}(\kappa_{RF} R_{RF})^2} \tag{5}$$

where ϵ_{RF} is the RF permittivity, *i.e.* the permittivity of the solvent, κ_{RF} is the inverse Debye screening length, usually set to 0 for explicit solvent MD and R_{RF} is the RF cut-off. Eqs. (3) and (4) are known as the distance dependent and independent terms, respectively. Eq.(2) is not evaluated for excluded atoms (those connected by 4 or less atoms, *e.g.* a torsion angle), while Eqs. (3) and (4) are evaluated for these atoms, as well.

Given the long-range nature of Eqs. (2), (3) and (4), a raw Residue Interaction Network (RIN) is rather impractical for visualization purposes. We have adopted two strategies to alleviate this issue: an interaction is considered only if it is above $k_B T$, where k_B is the Boltzmann constant and T is the temperature in kelvin; all interactions are consolidated at the residue level, in other words a node, *i.e.*, an AA, can have multiple edges *i.e.* an interaction, based on it constituent charge-groups (see Fig. 8).

2 Performance

In this section reports the time consumed by the RIP-MD jobs used as examples in the main text. The following tables describe features of the job (Table 1) and time to execute each of the parts of the job (Table 2 for operations over files and Table 3 for time consumed to compute each interaction type). These calculations

were performed on a PC with 2 Intel Xeon E5-2690 v3 CPU (each 12 cores with 24 threads) and 126 GB of RAM.

System	# of Atoms	# of Frames	# of cores used
MD2 (open)	2267	1250	20
MD2 (closing)	2267	11250	20
MD2 (closed)	2267	8618	20
GJC	42708	17589	40

Table 1: Number of atoms and frames for MD2 in separated in its three windows and the conexin 26 GJC. The third column reports the number of cores used to determine RINs.

System	Extracting frames	Writing attributes	Saving graphs
MD2 (open)	0:00:07	0:00:08	0:02:22
MD2 (closing)	0:01:23	0:01:09	0:21:47
MD2 (closed)	0:01:03	0:00:51	0:28:55
GJC	0:55:45	0:14:29	1:07:29

Table 2: Time in h:mm:ss format employed to perform each task on the MD trajectories of MD2 in separated in its three windows and the conexin 26 GJC.

System	Cα	H Bonds	Salt bridge	Cation -π	π-π	Arg- Arg	vdW	Coulomb	Correlation
MD2 (open)	0:00:08	0:01:26	0:00:04	0:00:06	0:00:06	0:00:03	1:04:30	0:52:25	0:08:43
MD2 (closing)	0:01:01	0:13:13	0:00:27	0:00:48	0:00:57	0:00:21	9:33:10	8:09:51	1:13:17
MD2 (closed)	0:00:46	0:09:57	0:00:21	0:.00:35	0:00:43	0:00:17	7:22:40	7:22:40	1:00:34
GJC	2:43:41	3:44:27	0:11:57	2:10:09	4:08:28	0:09:58	N/A	N/A	N/A

Table 3: Time in h:mm:ss format employed to determine each type of interaction. N/A indicates absence of the corresponding type of data for the condition.

3 Comparison of RIP-MD with other methods

There are several programs or libraries that generate RINs from static structures and/or for MD trajectories (summarized in table 4. In order to provide a comparison between the functionalities of RIP-MD and those of other available tools, we run Carma [14] and MD-TASK [6] on our first example, the closure of MD2 pocket. These two programs were chosen due to their availability, they are easy to install and they consider both inter and intra chain interactions to build RINs from MD simulations.

While Carma was designed to study Contact Maps (CMs) between C_α atoms of AAs, MD-TASK generates RINs only using contacts between C_β. Neither of these progrmas generates a consensus RIN, allowing only for a straight comparsion between their respective resulting CMs for each snapshot of the MD simulation. Nonetheless, we developed two simple scripts that generate a consensus RIN, one for the output of each approach, which solely keep those interactions that are present in at least 75% of the simulated time. Both scripts

can be downloaded from `https://github.com/Cold7/consensus_rin`. The consensus RINs generated by these two scripts and the equivalent consensus RIN obtained with C_α atoms by RIP-MD are shown in Fig. 9. RIP-MD and Carma generate identical networks, as it was expected, but the comparison with MD-TASK indicates a distinct, but similar response between the two different approaches to construct RINs.

When the same RINs are generated using non-covalent interactions using RIP-MD (Fig. 10), they show a similar behaviour as with the other definitions (contancts between C_αs or between C_βs). Nevertheless, the usage of non-covalent interactions allows to identify those chemical groups within the protein structure that are key to maintain the structure in each step, and in doing so, add more detail to the analysis of the MD simulation.)

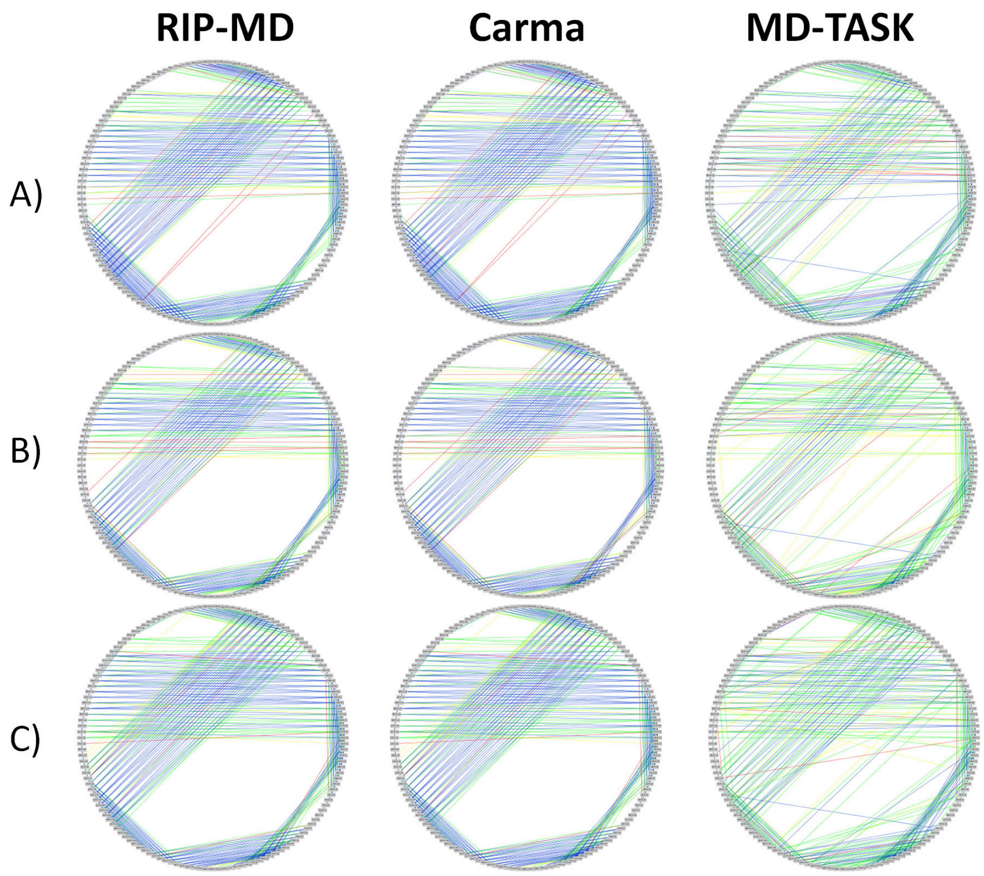

Figure 9: RINs generated by RIP-MD, Carma and MD-TASK for our MD2 example. A) CM for the open state; B) CM for the closing state; C) CM for the closed satea, each computed with each software. While RIP-MD and Carma show the same number of edges (591, 567 and 597 for each window), MD-TASK show 523, 516 and 550 edges for each window. Edge colors are described as follow. Red: Contacts existing in [75% - 80%) of the simulated time. Yellow: Contacts existing in [80% - 90%). Green: Contacts existing in [90% - 100%). Blue: Contacts existing in all simulated time.

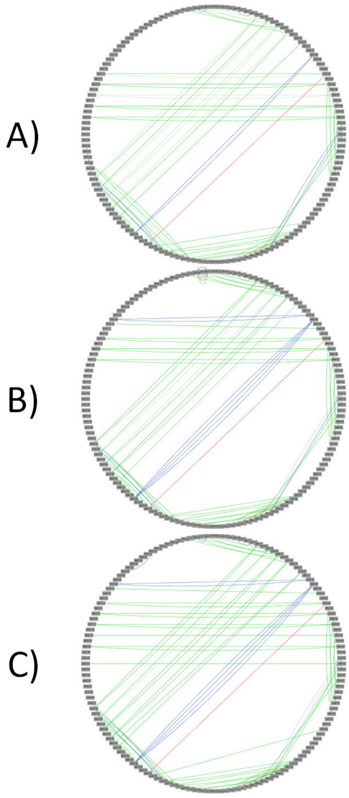

Figure 10: RINs generated by RIP-MD from the MD2 example. The networks shown correspond to the same system as in Fig. 9 but made with HBs (green edges), π-π interactions in red and slat bridges in blue, all present in at least 75% of the simulation. C_α contacts, VdW interactions and Coulomb contacts were omitted to highlight diferences with other methods. A) CM for the open. B) CM for the closing. C) CM for closed state.

Program / Library	Description	Reference
MDAnalysis	Python library to analyze trajectories from MD simulations. MDAnalysis implements atom selection commands, allowing to work with atomic coordinates, and thus, to compute c_α contacts.	[24]
MD-TASK	Suite of Python scripts that have been developed to analyze molecular dynamics trajectories. These scripts fall into 3 categories: Residue Interaction Networks, Perturbation analysis Response Scanning and Dynamic Cross-Correlation	[6]
PROTMAP2D	Tool for calculation, visualization and comparison of contact maps. It can be used for quantitative and qualitative characterization of differences and similarities between alternative models of the same protein, e.g. crystal structures solved under different conditions and/or series of conformations obtained in the course of MD simulations.	[27]
MDcons	Analysis of conserved contacts during MD simulations of Protein, RNA, DNA and Ligand based complexes. The input is either a MD trajectory, a set of snapshots or a single snapshot. The outputs are (1) maps of most/less frequently conserved contacts during MD or (2) a list of most/less frequently conserved contacts during MD.	[1]
ConAn	Tool to analyze MD trajectories through the use of contact maps and related quantities. It can be very useful for a first, exploratory analysis, i.e., identifying key contacts to be analyzed further, but can also offer alternative, possibly more precise, measures to the ones commonly used (RMSD, RMSF, etc...)	[22]
carma	CA-CA distance map calculator, but it also supports most of the steps required for a principal component analysis of molecular dynamics trajectories (also known as essential dynamics analysis).	[14]

Table 4: Programs and libraries that can compute RINs from MD simulations.

References

[1] S. Abdel-Azeim, E. Chermak, A. Vangone, R. Oliva, and L. Cavallo. Md-cons: Intermolecular contact maps as a tool to analyze the interface of protein complexes from molecular dynamics trajectories. *BMC Bioinformatics*, 15(5):S1, 2014.

[2] D. Alewood, K. Nielsen, P. F. Alewood, D. J. Craik, P. Andrews, M. Nerrie, S. White, T. Domagala, F. Walker, J. Rothacker, A. W. Burgess, and E. C. Nice. The role of disulfide bonds in the structure and function of murine epidermal growth factor (megf). *Growth Factors*, 23(2):97–110, 2005.

[3] E. V. Anslyn and D. A. Dougherty. *Modern Physical Organic Chemistry*, volume 69. 2006.

[4] R. B. Best, X. Zhu, J. Shim, P. E. M. Lopes, J. Mittal, M. Feig, and A. D. MacKerell. Optimization of the additive charmm all-atom protein force field targeting improved sampling of the backbone ϕ, ψ and side-chain $\chi 1$ and $\chi 2$ dihedral angles. *Journal of Chemical Theory and Computation*, 8(9):3257–3273, 2012.

[5] H. R. Bosshard, D. N. Marti, and I. Jelesarov. Protein stabilization by salt bridges: concepts, experimental approaches and clarification of some misunderstandings. *Journal of Molecular Recognition*, 17(1):1–16, 2004.

[6] D. K. Brown, D. L. Penkler, O. S. Amamuddy, C. Ross, A. R. Atilgan, C. Atilgan, and Ö. T. Bishop. MD-TASK: a software suite for analyzing molecular dynamics trajectories. *Bioinformatics (Oxford, England)*, 27, 2017.

[7] W. D. Cornell, P. Cieplak, C. I. Bayly, I. R. Gould, K. M. Merz, D. M. Ferguson, D. C. Spellmeyer, T. Fox, J. W. Caldwell, and P. A. Kollman. A second generation force field for the simulation of proteins, nucleic acids, and organic molecules. *Journal of the American Chemical Society*, 117(19):5179–5197, 1995.

[8] A. A. Dombkowski, K. Z. Sultana, and D. B. Craig. Protein disulfide engineering. {*FEBS*} *Letters*, 588(2):206 – 212, 2014.

[9] J. E. Donald, D. W. Kulp, and W. F. DeGrado. Salt bridges: Geometrically specific, designable interactions. *Proteins: Structure, Function, and Bioinformatics*, 79(3):898–915, 2011.

[10] D. A. Dougherty. Cation$-\pi$ interactions involving aromatic amino acids. *The Journal of Nutrition*, 137(6):1504S–1508S, 2007.

[11] J. Eargle and Z. Luthey-Schulten. Networkview: 3d display and analysis of protein·rna interaction networks. *Bioinformatics (Oxford, England)*, 28(22):3000–1, 2012.

[12] J. P. Gallivan and D. A. Dougherty. Cation-π interactions in structural biology. *Proceedings of the National Academy of Sciences*, 96(17):9459–9464, 1999.

[13] J. Glasgow, T. Kuo, and J. Davies. Protein structure from contact maps: A case-based reasoning Approach. *Information Systems Frontiers*, 8(1):29–36, 2006.

[14] N. M. Glykos. Software news and updates carma: A molecular dynamics analysis program. *Journal of Computational Chemistry*, 27(14):1765–1768.

[15] P. A. Hunt, C. R. Ashworth, and R. P. Matthews. Hydrogen bonding in ionic liquids. *Chem. Soc. Rev.*, 44:1257–1288, 2015.

[16] S.-M. Liao, Q.-S. Du, J.-Z. Meng, Z.-W. Pang, and R.-B. Huang. The multiple roles of histidine in protein interactions. *Chemistry Central Journal*, 7(1):1–12, 2013.

[17] A. MacKerell, D. Bashford, M. Bellot, R. Dunbrack, J. Evanseck, M. Field, S. Fischer, J. Gao, H. Guo, S. Ha, D. Joseph-McCarthy, L. Kuchnir, K. Kuczera, F. Lau, C. Mattos, S. Michnick, T. Ngo, D. Nguyen, B. Prodhom, W. Reiher, B. Roux, M. Schlenkrich, J. Smith, R. Stote, J. Straub, M. Watanabe, J. Wiorkiewicz-Kuczera, D. Yin, and M. Karplus. All-Atom Empirical Potential for Molecular Modeling and Dynamics Studies of Proteins. *Journal of Physical Chemistry B*, 102(18):3586–3616, 1998.

[18] A. D. MacKerell, D. Bashford, M. Bellott, R. L. Dunbrack, J. D. Evanseck, M. J. Field, S. Fischer, J. Gao, H. Guo, S. Ha, D. Joseph-McCarthy, L. Kuchnir, K. Kuczera, F. T. K. Lau, C. Mattos, S. Michnick, T. Ngo, D. T. Nguyen, B. Prodhom, W. E. Reiher, B. Roux, M. Schlenkrich, J. C. Smith, R. Stote, J. Straub, M. Watanabe, J. Wiórkiewicz-Kuczera, D. Yin, and M. Karplus. All-atom empirical potential for molecular modeling and dynamics studies of proteins. *The Journal of Physical Chemistry B*, 102(18):3586–3616, 1998.

[19] A. D. MacKerell, M. Feig, and C. L. Brooks. Improved treatment of the protein backbone in empirical force fields. *Journal of the American Chemical Society*, 126(3):698–699, 2004.

[20] A. Magalhaes, B. Maigret, J. Hoflack, J. N. F. Gomes, and H. A. Scheraga. Contribution of unusual arginine-arginine short-range interactions to stabilization and recognition in proteins. *Journal of Protein Chemistry*, 13(2):195–215, 1994.

[21] A. J. M. Martin, M. Vidotto, F. Boscariol, T. Di Domenico, I. Walsh, and S. C. E. Tosatto. Ring: networking interacting residues, evolutionary information and energetics in protein structures. *Bioinformatics (Oxford, England)*, 27(14):2003–5, 2011.

[22] D. Mercadante, F. Gräter, and C. Daday. Conan: A tool to decode dynamical information from molecular interaction maps. *Biophysical Journal*, 114(6):1267 – 1273, 2018.

[23] E. A. Meyer, R. K. Castellano, and F. Diederich. Interactions with aromatic rings in chemical and biological recognition, 2003.

[24] N. Michaud-Agrawal, E. J. Denning, T. B. Woolf, and O. Beckstein. Md-analysis: A toolkit for the analysis of molecular dynamics simulations. *Journal of Computational Chemistry*, 32(10):2319–2327, 2011.

[25] M. A. C. Neves, M. Yeager, and R. Abagyan. Unusual arginine formations in protein function and assembly: Rings, strings, and stacks. *The Journal of Physical Chemistry B*, 116(23):7006–7013, 2012.

[26] C. Oostenbrink, A. Villa, A. E. Mark, and W. F. van Gunsteren. A biomolecular force field based on the free enthalpy of hydration and solvation: the gromos force-field parameter sets 53a5 and 53a6. *Journal of computational chemistry*, 25(13):1656–76, 2004.

[27] M. J. Pietal, I. Tuszynska, and J. M. Bujnicki. Protmap2d: visualization, comparison and analysis of 2d maps of protein structure. *Bioinformatics*, 23(11):1429–1430, 2007.

[28] T. Steiner. The hydrogen bond in the solid state. *Angewandte Chemie International Edition*, 41(1):48–76, 2002.

[29] W. F. van Gunsteren, P. H. Hünenberger, A. E. Mark, P. E. Smith, and I. G. Tironi. Computer simulation of protein motion. *Computer Physics Communications*, 91(1-3):305–319, 1995.

[30] J. Vondrášek, P. E. Mason, J. Heyda, K. D. Collins, and P. Jungwirth. The molecular origin of like-charge arginine-arginine pairing in water. *The Journal of Physical Chemistry B*, 113(27):9041–9045, 2009.

[31] J. Wang, H. Ma, X. Ge, J. Zhang, K. Teng, Z. Sun, and J. Zhong. Bovicin hj50-like lantibiotics, a novel subgroup of lantibiotics featured by an indispensable disulfide bridge. *PLoS ONE*, 9(5):1–11, 2014.

[32] Z. Wang and J. Xu. Predicting protein contact map using evolutionary and physical constraints by integer programming. *Bioinformatics*, 29(13):266–273, 2013.

[33] Z. Wang and J. Xu. Predicting protein contact map using evolutionary and physical constraints by integer programming. *Bioinformatics*, 29(13):i266, 2013.

[34] M. L. Williams and J. E. Gready. Guanidinium-type resonance stabilization and its biological implications. i. the guanidine and extended-guanidine series. *Journal of Computational Chemistry*, 10(1):35–54, 1989.
